# GPR132 regulates the function of NK cells through the Gαs/CSK/ZAP70/NF-κB signaling pathway as a potential immune checkpoint

**DOI:** 10.1126/sciadv.adr9395

**Published:** 2025-03-05

**Authors:** Xinhui Hui, Min Xue, Yaojun Ren, Yiran Chen, Xuenan Chen, Muhammad Asad Farooq, Yuzhou Ji, Weirong Zhan, Yunhe Huang, Bingtan Du, Jie Yao, Yixin Duan, Wenzheng Jiang

**Affiliations:** ^1^ Shanghai Key Laboratory of Regulatory Biology, School of Life Sciences, East China Normal University, Shanghai, China.; ^2^ College of Life Science, Xinjiang Normal University, Urumqi, China.; ^3^ The First Dongguan Affiliated Hospital, Guangdong Provincial Key Laboratory of Medical Immunology and Molecular Diagnostics, Guangdong Medical University, Dongguan, China.

## Abstract

As a member of the proton-sensing GPCR receptors, GPR132 plays a crucial role in regulating immune cell functions, but the mechanism by which GPR132 affects natural killer (NK) cells has not yet been reported. Here, RNA-seq displayed that the expression of GPR132 was reduced in activated NK cells, and the proportion of mature NK cells in GPR132^−/−^ mice was substantially increased compared to WT mice, with stronger anti-melanoma capabilities. Further investigation indicates that GPR132-deficient NK92 cells expressed more GzmB and IFN-γ and exhibited stronger cytotoxicity. Mechanically, GPR132 regulates NK cell function through the CSK/ZAP70/NF-κB signaling axis. Down-regulation of GPR132 weakens the inhibition of NK cell function by lactate, thereby enhancing the functional execution of CAR-NK cells against colorectal cancer. These results highlight the previously unrecognized role of GPR132 in the regulation of NK cell function and that inhibition of GPR132 provided an updated insight for NK cell therapy.

## Teaser

GPR132 exhibits an inhibitory effect on the antitumor capabilities of NK cells via the CSK/ZAP70/NF-κB signaling axis.

## INTRODUCTION

The G protein–coupled receptor (GPCR) family is the largest family of membrane receptors, with more than 800 members (*1*). GPCRs have a seven-transmembrane domain and play a crucial role in regulating physiological and pathological processes (*2*). Around 35% of marketed drugs achieve therapeutic effects through GPCRs, making them attractive drug targets (*3*, *4*). There is a class of GPCRs that can sense an extracellular acidic environment and are activated at low pH to regulate physiological processes such as cell growth, metabolism, and migration. These include GPR4, GPR65 (TDAG8), GPR68 (OGR1), and GPR132 (G2A) (*5*, *6*). GRR132 is the most abundant proton-sensing GPCR in immune cells and is involved in migration, proliferation, and differentiation (*7*–*10*). It has been reported apoptosis and differentiation in acute myeloid leukemia (AML) cells can be inducible by activating GPR132 using ONC212 and 8-GL (*7*, *11*). In the breast cancer microenvironment, GPR132 inhibitors interfere with the lactate-GPR132-peroxisome proliferator–activated receptor γ axis and encourage tumor-associated macrophages to the M1-phenotype transformation (*12*). Furthermore, antagonists of GPR132 can alter macrophage reprogramming within pancreatic islets, thereby improving diabetes (*13*). These studies suggest that GPR132 is a potential immunotherapy checkpoint.

Natural killer (NK) cells are a type of innate immune cell that plays a crucial role in controlling malignancy and infections. In contrast to T cells, NK cells are able to target MHC (or HLA) molecule-deficient tumor cells, induce apoptosis by releasing cytotoxic perforins and granzymes, and do not cause immune rejection in adoptive cell therapy (*14*). Therefore, NK cells have great potential in antitumor immune cell therapy. Despite this, NK cells are still not widely used in the clinical treatment of solid tumors. On the one hand, solid tumors are difficult to reach and infiltrate (*15*), and on the other hand, the main obstacle is the hypoxic tumor microenvironment (TME) that produces large amounts of lactate through metabolism, leading to NK cell depletion (*16*, *17*). Recently, most studies have focused on using immunostimulants, immune checkpoint blockade (IBD), and chimeric antigen receptor (CAR) to enhance NK cell antitumor activity and specificity (*18*).

In our research, we demonstrated that GPR132 affects NK cell function through the Gαs/C-terminal Src kinase (CSK)/ZAP70/nuclear factor κB (NF-κB) signaling pathway. In addition, the down-regulation of GPR132 in NK cells weakens the inhibition by lactate and substantially enhances the effectiveness of CAR-NK92 cells against colorectal cancer. In summary, our study uncovers the regulatory mechanism of GPR132 on NK cells, aiming to reduce the immunosuppression of CAR-NK cells in the solid TME by down-regulating GPR132, which offers valuable insights for cell therapy.

## RESULTS

### The expression of GPR132 decreases after NK cell activation

**Fig. 1.**
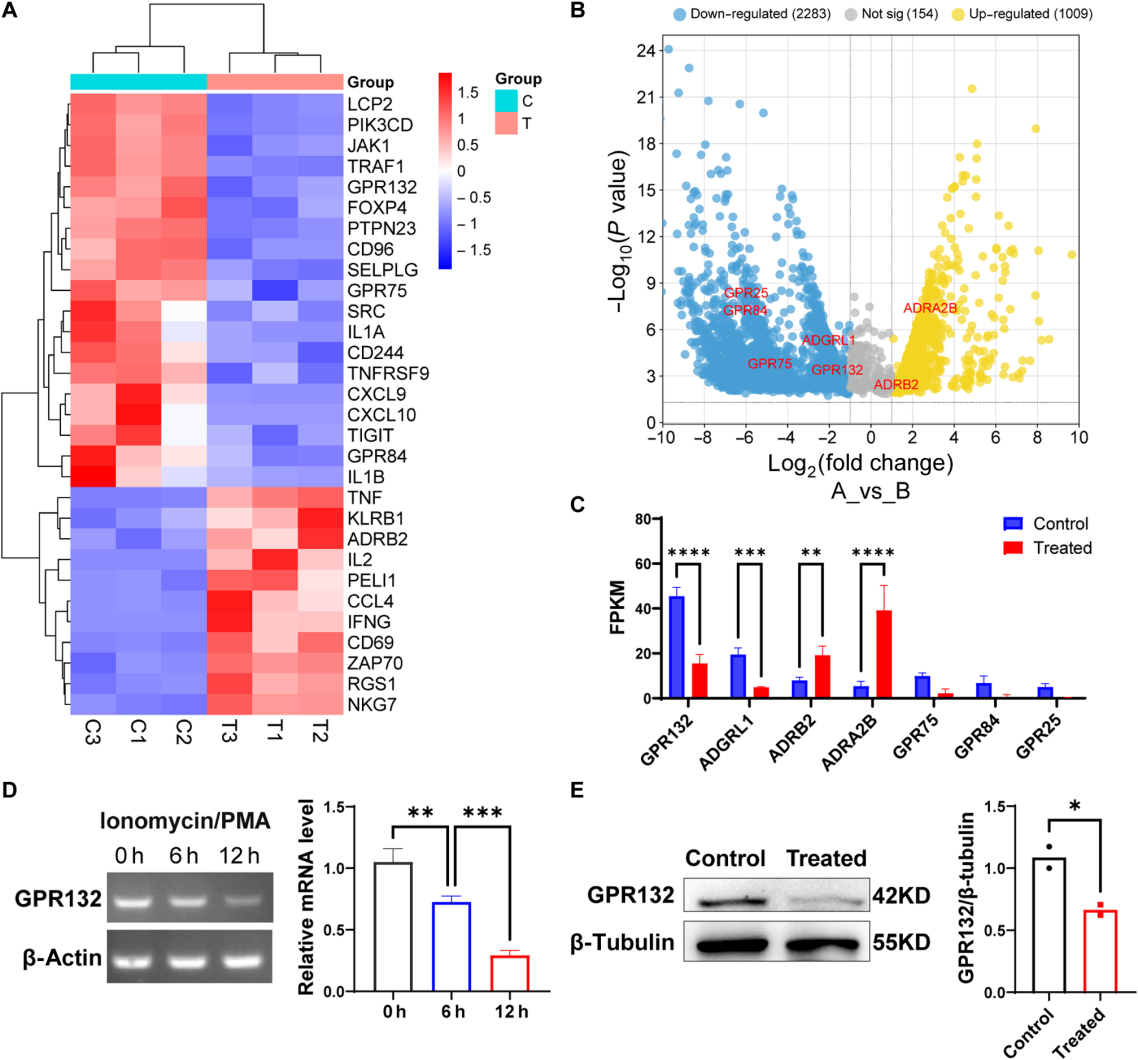
The expression of GPR132 decreased in activated NK cells. (**A** to **C**) RNA-seq analysis of gene expression in activated NK cells compared with a control group (*n* = 3). (A) Heatmap shows the expression variation of NK activation–related markers and inhibition of NK activation markers. (B) Volcano plot shows 3446 genes shared by three donors. Down-regulated genes, blue; up-regulated genes, yellow; interested genes, red. (C) Column chart shows the expression of the interested GPCR gene according to FPKM value. (**D**) RT-PCR to detect the GPR132 expression in NK92 cells after being stimulated by ionomycin (1 μg/ml) and PMA (20 ng/ml) at 0, 6, or 12 hours. β-Actin was used as a reference housekeeping gene (*n* = 3, biological replicates). (**E**) WB determined the GPR132 expression in NK92 cells after being stimulated by ionomycin (1 μg/ml) and PMA (20 ng/ml) for 12 hours. β-Tubulin was used as a reference housekeeping gene. Data represent means ± SD. Analyzed by two-way analysis of variance (ANOVA), one-way ANOVA, or unpaired *t* test (*P < 0.05, **P < 0.01, ***P < 0.001, and ****P < 0.0001).

First, we isolated primary NK cells from human peripheral blood mononuclear cells (PBMCs). The treatment group was stimulated with ionomycin and phorbol 12-myristate 13-acetate (PMA), and the untreated cells were used as control. After 6 hours of activation, the samples were collected, and RNA sequencing (RNA-seq) analysis was performed. The RNA-seq revealed 3446 differentially expressed genes shared by the three donors, most of which were associated with biological processes (fig. S1, A and B). The heatmap displays a notable up-regulation of NK cell activation markers, including tumor necrosis factor (TNF), CD69, and interferon-γ (IFN-γ). In contrast, negative regulatory genes, such as TIGIT and CD244, were notably down-regulated (Fig. 1A). In addition, Kyoto Encyclopedia of Genes and Genomes (KEGG) analysis revealed that NF-κB and other related pathways were enriched following NK cell activation (fig. S1C). The volcano map of the differential genes showed notable changes in the expression levels of certain GPCRs of interest (Fig. 1B). Statistical analysis revealed that the GPR132, a member of the proton-sensing GPCR family, was mostly significantly down-regulated (Fig. 1C). Furthermore, we stimulated the NK92 cell line with ionomycin and PMA; collected the cells at 0, 6, and 12 hours to extract RNA; and performed reverse transcription polymerase chain reaction (RT-PCR) and Western blot (WB). Consistent with the RNA-seq results, the expression of GPR132 decreased profoundly in activated NK92 cells (Fig. 1, D and E). Consequently, we hypothesized that GPR132 may be related to NK cell function regulation.

### Deletion of GPR132 enhances the anti-melanoma function of NK cells in mice

**Fig. 2.**
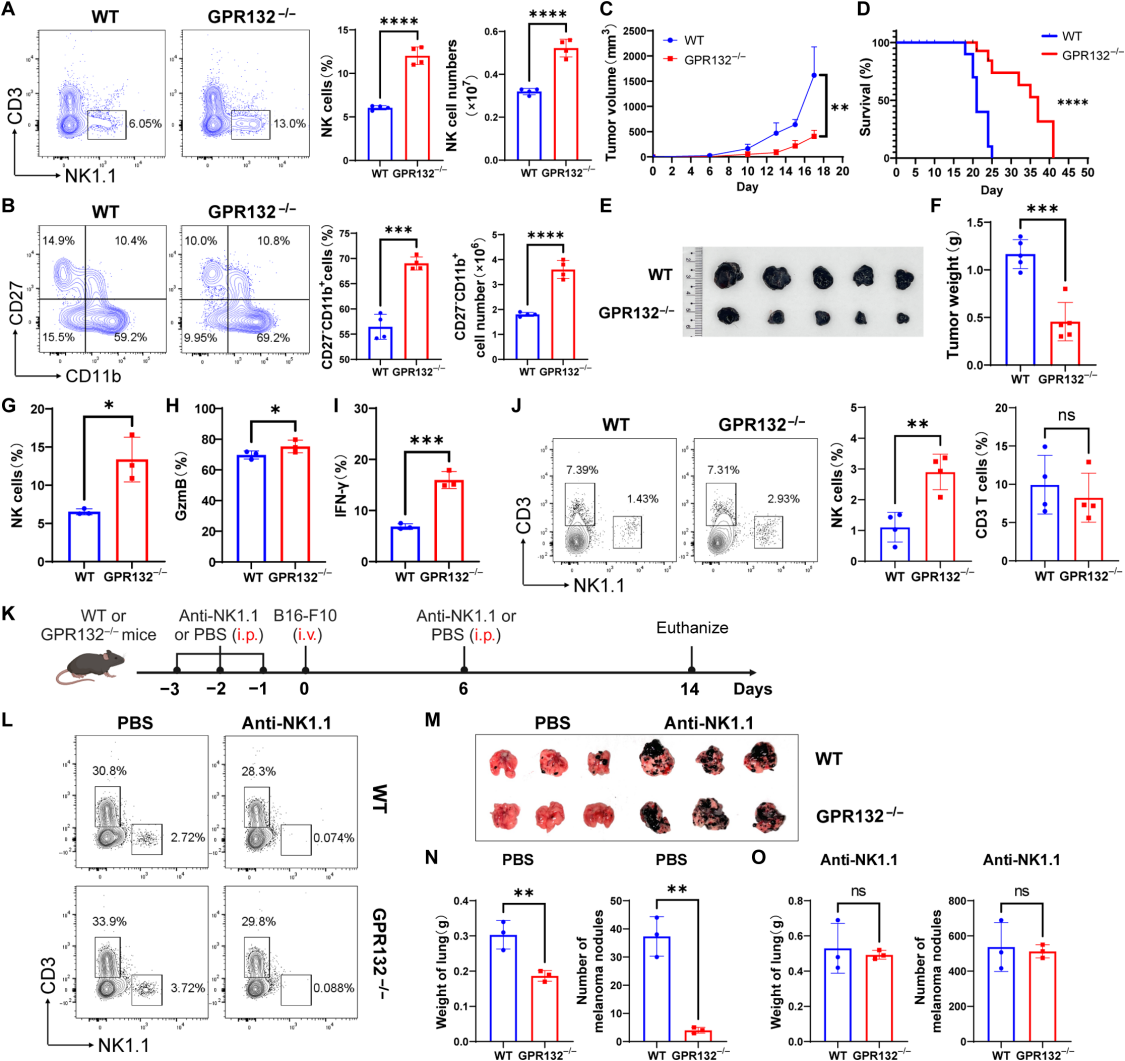
Knockout of GPR132 enhanced the ability of NK cells to resist melanoma. (**A** and **B**) FACS analysis of the NK cells (A) and CD27^−^CD11b^+^ NK cells (B) percentage in the spleen (*n* = 4). The representative FACS profile is on the left, and the column chart is on the right. (**C**) Tumor volume was measured every 2 days, and growth curves were shown (*n* = 5). (**D**) Kaplan-Meier survival curve visually depicted the overall survival rate for WT and GPR132^−/−^ mice in a 50-day survival study (*n* = 10). (**E** to **I**) Melanomas were divested when the tumor volume reached 1500 to 2000 mm^3^, the tumors were photographed (E) and weighted (F) (*n* = 5), then the proportion of NK cells inside the tumors (G) and the expression of GzmB (H) and IFN-γ (I) in tumor-infiltrating NK cells (*n* = 3) were analyzed by FACS. (**J**) FACS analysis of the NK cell proportion in the peripheral blood of recipients after receiving WT and GPR132^−/−^ bone marrow cells for 4 weeks. (**K**) Schematic diagram of melanoma lung metastasis experiment. On day 0, 3 × 10^5^ B16-F10 cells were injected into per mouse via the tail vein. (**L**) On day 10, FACS analysis was performed to determine the NK cell depletion in the peripheral blood of anti-NK1.1 or PBS-treated mice. On day 14, the melanoma colony numbers were counted in the lungs and the weight of the lungs was measured (*n* = 3). (**M** to **O**) Photographs of lungs from the PBS and anti-NK1.1–treated group are shown at the top (M), and the statistical charts [(N) and (O)] are shown at the bottom. Data are shown as means ± SD and were analyzed by unpaired *t* test or log-rank test (*P < 0.05, **P < 0.01, ***P < 0.001, and ****P < 0.0001; ns, not significant).

NK cell proportions in different organs from GPR132^−/−^ mice and wild-type (WT) mice were analyzed by flow cytometry. The results showed that the percentage of NK cells in the peripheral blood of GPR132^−/−^ mice increased significantly compared to WT mice, while the proportion of T cells remained unchanged (fig. S2). We further observed a considerable increase in splenic NK cells of GPR132 deficient mice (Fig. 2A), manifested by an increase in the percentage and number of CD27^−^CD11b^+^ NK population (Fig. 2B), which was identified as mature NK cells with strong lysis capacity (*19*). However, there were no notable proportion differences in other immune cells present in the spleen (fig. S3, A to D). In addition, a moderate increase in NK cells was noted in the liver, lung, and bone marrow of GPR132-deficient mice (fig. S4, A to D). Afterward, we injected 5 × 10^5^ B16-F10 cells into WT and GPR132^−/−^ mice subcutaneously to establish a melanoma-bearing model. As per the findings, the tumor volume and weight bore with GPR132^−/−^ mice was notably lower than in WT mice (Fig. 2, C, E, and F). In addition, compared to WT mice, GPR132-deficient mice exhibited a higher survival rate (Fig. 2D), an elevated number of tumor-infiltrating NK cells (Fig. 2G), which expressed more granzyme B (GzmB) (Fig. 2H) and IFN-γ (Fig. 2I). The results above suggest that GPR132 may affect the maturation of NK cells and thus the antitumor ability of mice, but we cannot rule out ILC1s from this NK1.1 population, especially in the liver and the lung.

To further verify whether the inhibitory of GPR132 on NK cell homeostasis, we transplanted bone marrow cells from WT and GPR132^−/−^ mice into WT mice irradiated with lethal doses (fig. S5A) and observed NK cell proportion and antitumor activity in recipients. Consistent with previous results, the mice that received GPR132^−/−^ bone marrow cells exhibited a higher proportion of NK cells in peripheral blood and inside the tumor as well as showed stronger resistance to melanoma compared to the WT group (Fig. 2J and fig. S5, B and C). Moreover, to further explore whether GPR132 deletion could promote the ability of NK cells to resist tumor metastasis, we conducted a melanoma lung metastasis experiment (Fig. 2K). We found that melanin colonies metastasized in the lungs of GPR132^−/−^ mice were lower than in WT mice, and this difference was abolished with the administration of anti-NK1.1 (Fig. 2, L to O). The findings reveal that GPR132 deletion promotes the antitumor ability of mice through NK cells.

### The deficiency of GPR132 enhances the cytotoxicity and activation of NK cells

**Fig. 3.**
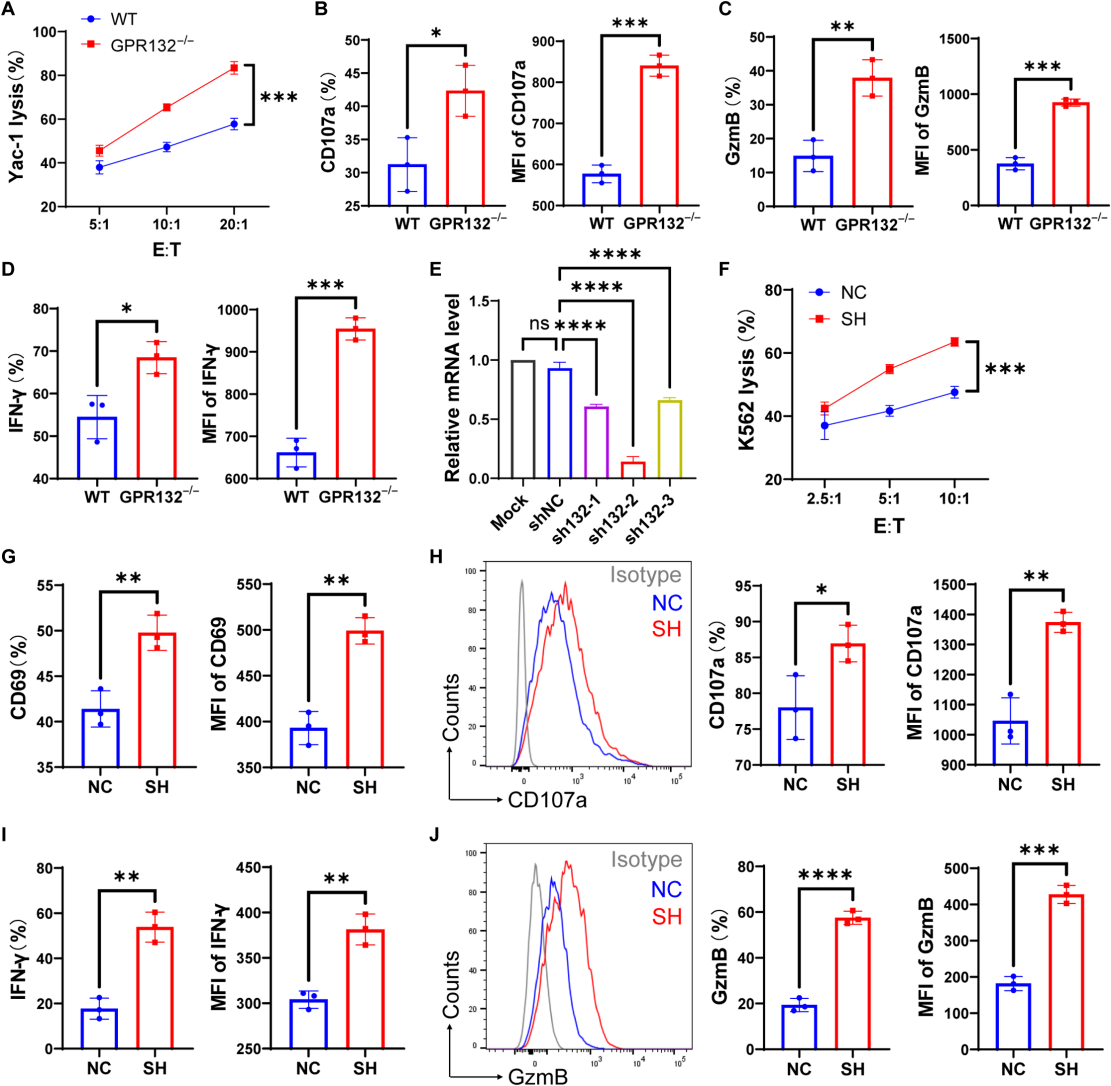
Deficiency of GPR132 improves the cytotoxicity of NK cells. (**A**) The line plots displayed the killing ability of WT and GPR132^−/−^ mice splenic NK cells against Yac-1 cells for 4 hours. The E:T ratios are 5:1, 10:1, and 20:1. (**B** to **D**) FACS analysis of CD107a, GzmB, and IFN-γ expression in splenic NK cells of WT and GPR132^−/−^ mice after coculture with target cells at a 10:1 ratio for 4 hours. (**E**) NK92 cells were infected with Mock, shNC, and shRNA lentivirus for 48 hours and then collected to perform an RT-PCR analysis to verify the interference efficiency of GPR132. The multiplicity of infection (MOI) is 10. (**F**) Line plots illustrate the killing ability of NK92 cells against K562 cells at various E:T ratios over a 4-hour duration. NC was the NK92 cells infected with shRNA-NC lentivirus, and SH was the NK92 cells infected with GPR132-shRNA2 lentivirus at MOI = 10. (**G** to **J**) After coculture with K562 cells at a 5:1 (E:T) ratio for 4 hours, FACS was conducted to detect the expression of CD69 (G), CD107a (H), IFN-γ (I), and GzmB (J). For (H) and (J), the representative FACS histogram is displayed on the left, and the column chart is on the right. The experiment was performed at least three times. Data are shown as means ± SD. Analyzed by unpaired *t* test or one-way ANOVA (*P < 0.05, **P < 0.01, ***P < 0.001, and ****P < 0.0001). MFI, mean fluorescence intensity.

To further demonstrate the effect of GPR132 on NK function in vitro, we isolated splenic NK cells from both WT and GPR132^−/−^ mice and co-incubated with Yac-1 cells for 4 hours at different effector-to-target (E:T) ratios. GPR132 deletion resulted in an increased killing efficiency of NK cells, as shown in Fig. 3A. In addition, the expression of CD107a, GzmB, and IFN-γ in NK cells from GPR132^−/−^ mice increased considerably compared to WT mice (Fig. 3). To further clarify the effect of GPR132 on NK cells, we designed three GPR132–short hairpin RNA (shRNA) sequences and a negative control (NC) shRNA (shNC) sequence targeting the NK92 cell for in vitro functional validation (fig. S6, A and B). First, we verified that shRNA2 was the optimal sequence to knock down the GPR132 gene at transcriptional and protein levels by RT-PCR and WB (Fig. 3E and fig. S6, C and D) and applied it to subsequent experiments. Subsequently, the functionality of GPR132-interfering (SH) and non-interfering (NC) NK cells was evaluated after co-incubating with K562 cells for 4 hours. Our findings indicate that SH-NK92 cells exhibited stronger cytotoxicity against K562 cells at different E:T ratios compared to NC-NK92 cells (Fig. 3F and fig. S7) and expressed more CD69, CD107a, IFN-γ, and GzmB than NC-NK92 cells (Fig. 3, G to J), the similar results have been demonstrated with primary human NK cells (fig. S8, A to C). Briefly, these data indicate that the knockdown of GPR132 enhances the NK cell function.

### Activation of GPR132 inhibits the cytotoxicity and activation of NK cells

**Fig. 4.**
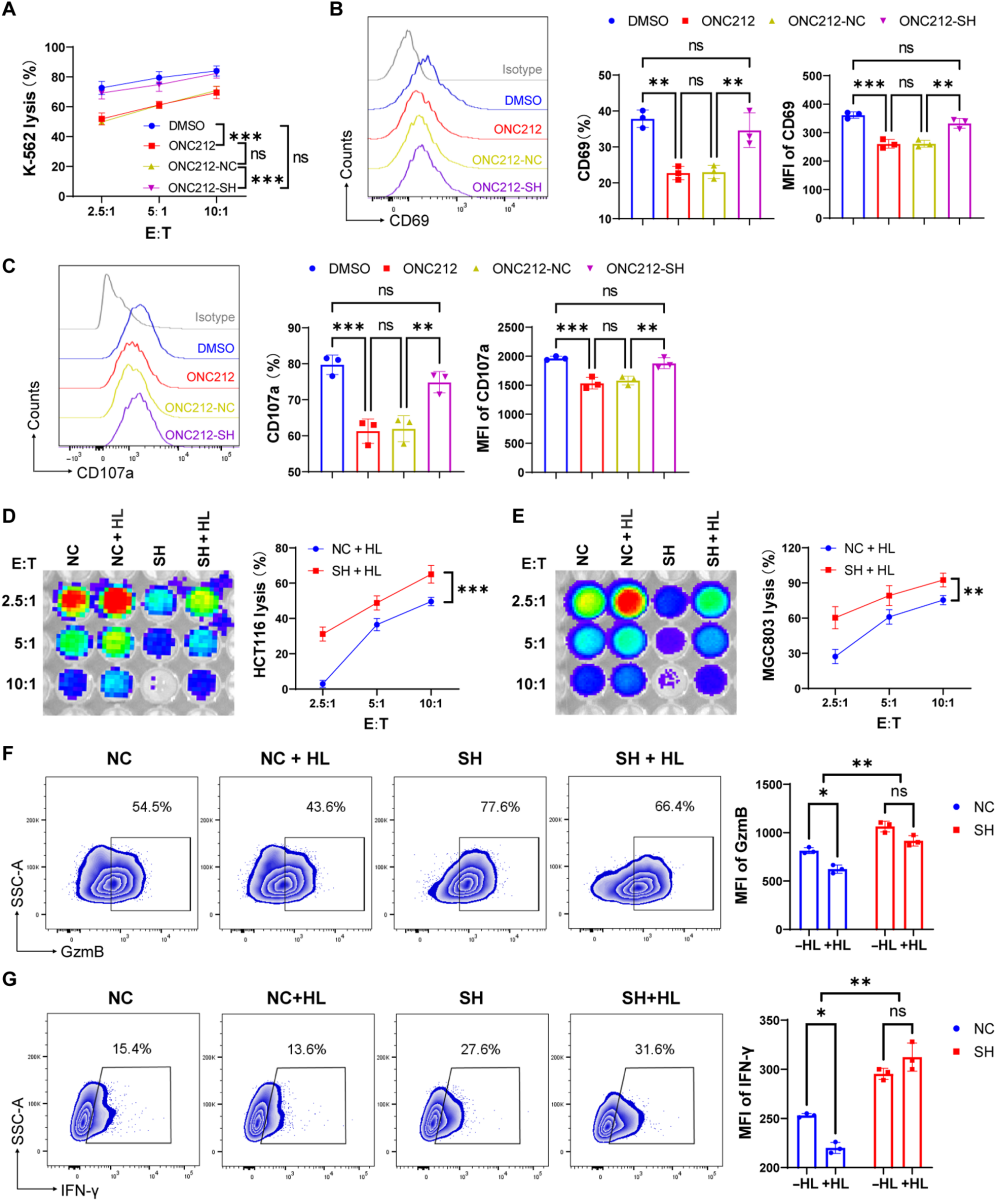
Activation of GPR132 inhibits the function of NK92 cells. (**A**) NK92 cells, NC-NK92 cells, and SH-NK92 cells were treated with DMSO or 5 μM ONC212 for 12 hours and cocultured with K562 cells at various E:T ratios for 4 hours to detect cytotoxicity. (**B** and **C**) Expression of CD107a and GzmB in the indicated group was analyzed by flow cytometry after coculture with K562 cells at a 5:1 (E:T) ratio for 4 hours. The representative FACS histogram is displayed on the left, and the column charts are on the right. (**D** and **E**) Lysis rate of HCT116-luciferase cells (D) and MGC803-luciferase cells (E) was detected by bioluminescence imaging after 4 hours cocultured with indicated NK92 group at different E:T ratios. NC + HL and SH + HL mean NK92 cells were treated with 10 mM lactate acid for 12 hours, with untreated NC-NK92 and SH NK92 cells as a control. (**F** and **G**) NC-NK92 and SH-NK92 cells were treated with or without 10 mM lactate for 12 hours and cocultured with HCT116 at a 5:1 (E:T) ratio for 4 hours, and subsequently, the expression of GzmB (F) and IFN-γ (G) were analyzed by flow cytometry. The representative FACS profile is displayed on the left, and the MFI column is on the right. Data are shown as means ± SD, and the experiment was performed three times. The experiment was performed three times. Data are shown as means ± SD. Analyzed by one-way ANOVA, unpaired *t* test, or two-way ANOVA (*P < 0.05, **P < 0.01, and ***P < 0.001).

ONC212 has been reported to be a selective agonist of GPR132 (*11*). To further verify that GPR132 has a negative impact on NK function, we conducted the following experiments. On the basis of the determination of ONC212 concentration in fig. S9, NK92 cells, NC-NK92 cells, and SH-NK92 cells were treated with dimethyl sulfoxide (DMSO) or ONC212 (5 μM) for 12 hours and subsequently cocultured with K562 at different E:T ratio for 4 hours to analyze cytotoxicity. Compared to the DMSO group, the killing efficiency of ONC212-treated NK92 cells was substantially reduced (Fig. 4A and fig. S10). Simultaneously, the expression of CD69 and CD107a was also decreased (Fig. 4, B and C). However, this effect was eliminated after GPR132 knockdown, demonstrating that ONC212 specifically activates GPR132 (Fig. 4, A to C, and fig. S10). However, this effect was eliminated after GPR132 knockdown, demonstrating that ONC212 specifically activates GPR132 (Fig. 4, A to C, and fig. S10). In addition, it is well known that a large amount of lactate produced by the TME inhibits the function of immune cells (*20*). We investigated whether lactate could have a similar effect to ONC212, given that GPR132 belongs to the pH-sensitive GPCR family and is supposed to be activated by lactate (*12*, *21*). Thus, NC-NK92 cells and SH-NK92 cells were treated with 10 mM lactate (HL) for 12 hours according to the determination of lactate concentration in fig. S11 and then co-incubated with HCT116 and MGC803 for 4 hours at different E:T ratios. The bioluminescence imaging displayed that lactate-treated SH-NK92 cells still maintained excellent killing capacity and higher activation levels than lactate-treated NC-NK92 cells (Fig. 4, D to G). In contrast with the NC-NK92 cohort, lactate-treated SH-NK92 exhibited no significant difference in GzmB and IFN-γ expression (Fig. 4, F and G) compared with untreated SH-NK92 cells. Our findings revealed that activation of GPR132 inhibited the function of NK cells, and GPR132 deficiency could weaken the inhibition of NK cells by lactate.

### GPR132 affects the proliferation and anti-apoptotic ability of NK cells

**Fig. 5.**
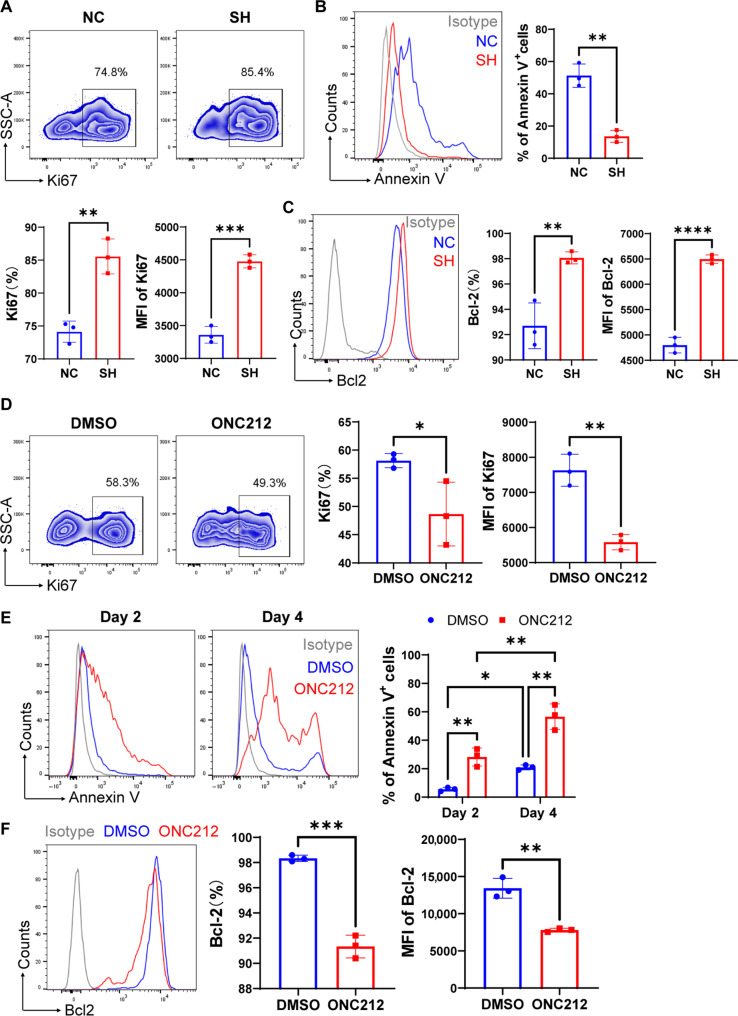
GPR132 affects the proliferation and apoptosis of NK92 cells. (**A** to **C**) NK92 cells were infected with shRNA-NC or shRNA-GPR132 lentivirus for 48 hours at MOI = 10 and cultured for 3 to 5 days. On day 5, the cells were collected to analyze Ki67 expression (A), and on day 4, annexin V^+^ cell proportion (B) and Bcl-2 expression (C) were analyzed by flow cytometry. (**D** to **F**) NK92 cells were treated with DMSO or ONC212 (5 μM) for 12 hours and cultured for continuous 2 to 5 days. FACS analysis was performed for Ki67 expression (D) on day 5, annexin V^+^ cell percentage (E) on days 2 and 4, and Bcl-2 expression (F) on day 4. For analysis of annexin V^+^ rate and Bcl-2 expression, NK92 cells were cultured without supplemented hIL-2. For (A), the representative FACS profile is shown at the top, and the column charts are at the bottom. For (B) to (F), the representative FACS histogram (left) and the column chart (right) are shown. The experiment was performed three times. Data are shown as means ± SD. Analyzed by unpaired *t* test or two-way ANOVA (*P < 0.05, **P < 0.01, ***P < 0.001, and ****P < 0.0001).

Previously, we noted that there was a noticeable increase in the proportion of NK cells in GPR132 knockout mice. As a result, we carried out additional validation using the human NK92 cells. We found that the knockdown of GPR132 resulted in a marked increase in the expression abundance of the proliferation antigen Ki67 (Fig. 5A). Furthermore, we cultured NC-NK92 cells and SH-NK92 cells without human recombinant interleukin-2 (hIL-2) to investigate their apoptosis and survival. The results revealed that, in comparison to the NC-NK92 cells, SH-NK92 cells presented reduced apoptosis (Fig. 5B), accompanied by a considerable increase in the expression of the anti-apoptotic protein Bcl-2 (Fig. 5C). Conversely, ONC212-treated NK92 cells displayed lower expression of Ki67 (Fig. 5D), increased apoptosis rates, and decreased Bcl-2 expression compared to the DMSO-treated NK92 cells (Fig. 5, E and F). These results indicated that GPR132 may regulate the proliferation and apoptosis of NK cells, and in the absence of IL-2, GPR132 deficiency can enhance the anti-apoptotic ability and survival of NK cells.

### GPR132 regulates NK cell function through the Gαs/CSK/ZAP70/NF-κB signal axis

**Fig. 6.**
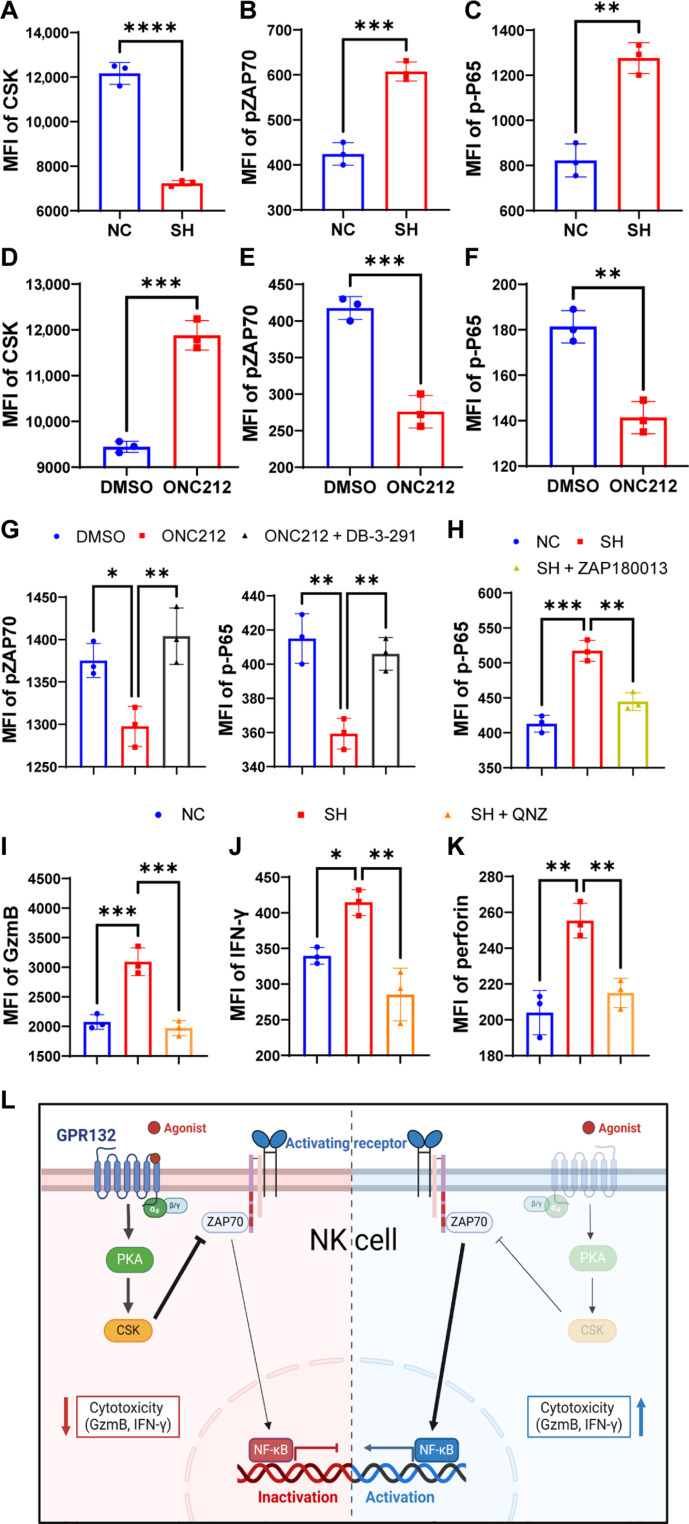
GPR132 inhibits the function of NK cells via the Gαs/CSK/ZAP70/NF-κB signal axis. (**A** to **C**) FACS analysis of the CSK, pZAP70, and p-P65 expression in NC-NK92 and SH-NK92 cells. (**D** and **F**) FACS analysis of the CSK, pZAP70, and p-P65 expression in DMSO-NK92 and ONC212-NK92 cells. CSK was stained by primary antibody at 4°C for 30 min and then stained with AF647 anti-rabbit IgG antibody. (**G**) DMSO-NK92 and ONC212-NK92 cells were treated with DMSO or 10 μM DB-3-291 (CSK degrader) to analyze the expression of pZAP70 and p-P65 by flow cytometry. (**H**) NC-NK92 and SH-NK92 cells were treated with DMSO or 5 μM ZAP180013 (ZAP70 inhibitor) to detect the expression of p-P65 by flow cytometry. (**I** to **K**) NC-NK92 and SH-NK92 cells were treated with DMSO or 5 μM QNZ (NF-κB inhibitor) to detect the expression of GzmB, IFN-γ, and perforin by flow cytometry. (**L**) Graphical abstract shows the summary of the signaling mechanism. The experiment was performed three times. Data are shown as means ± SD. Analyzed by unpaired *t* test or one-way ANOVA (*P < 0.05, **P < 0.01, ***P < 0.001, and ****P < 0.0001).

The heatmap in Fig. 1 shows that the ZAP70 gene is remarkably up-regulated with NK cell activation and enriched among the NF-κB pathway (fig. S1C), which is critical for NK function (*22*, *23*). It has been reported that ZAP70 is regulated by CSK in immune cells (*24*, *25*). Hence, we assessed the expression of CSK, pZAP70, and p-P65 in NK92 cells after down-regulation or activation of GPR132. We found that GPR132 positively regulated the expression of CSK but negatively regulated pZAP70 and p-P65 expression (Fig. 6, A to F, and fig. S12, A to F). To investigate whether CSK regulates the expression of pZAP70 and p-P65, we treated NK92 cells and ONC212-NK92 cells with DMSO or 10 μM CSK degrader, and the flow cytometry analysis was performed (fig. S13A). The results showed that the CSK degrader relieved the inhibition of pZAP70 and p-P65 expression caused by GPR132 activation (Fig. 6G and fig. S13, B and C), which indicates that CSK inhibits the ZAP70 and NF-κB pathway. We further treated NC-NK92 and SH-NK92 cells with DMSO or ZAP70 inhibitors to verify that ZAP70 certainly regulates the NF-κB pathway (Fig. 6H and fig. S14, A and B). Moreover, NF-κB inhibitor triggered a significant reduction in the expression of GzmB, IFN-γ, and perforin in SH-NK92 cells compared to the untreated group (Fig. 6, I to K, and fig. S15, A to D). We also demonstrated that GPR132 was positively correlated with the expression of Gαs but had no effect on Gαi1/2/3 (fig. S16, A, and B), which is consistent with a previous report (*7*). These results indicate that GPR132 regulates NK cell function through the Gαs/CSK/ZAP70/NF-κB signaling pathway (Fig. 6L).

### Down-regulation of GPR132 enhances the cytotoxicity and activation of CAR-NK cells

**Fig. 7.**
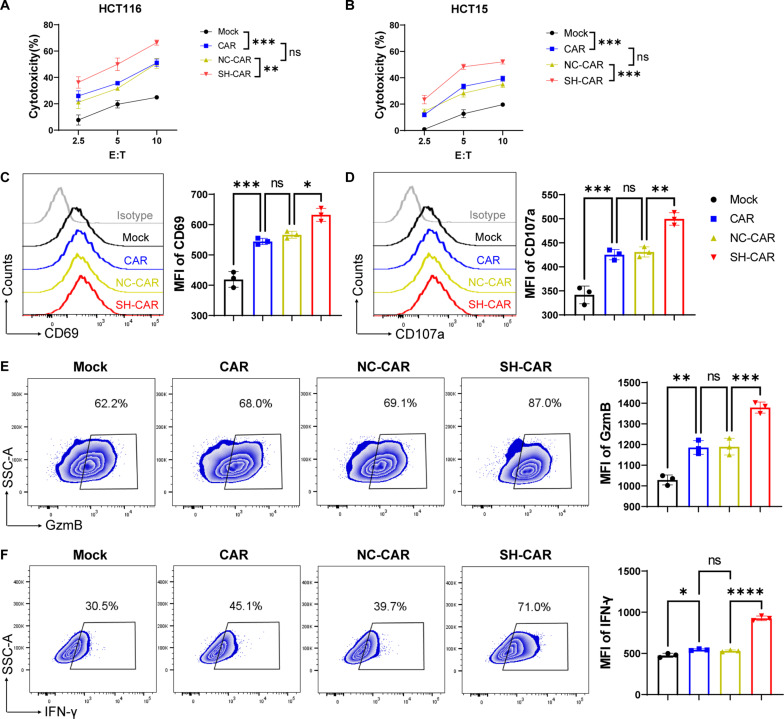
Down-regulation of GPR132 promotes the antitumor activity of CAR-NK92 cells in vitro. To prepare CAR-NK92 cells, 5 × 10^5^ NK92 cells were transduced with mock, CAR, NC-CAR, and SH-CAR lentiviruses, respectively, at MOI = 10. (**A** and **B**) Line plots displayed the killing ability of mock-NK92 cells, CAR-NK92 cells, NC-CAR-NK92 cells, and SH-CAR-NK92 cells against HCT116 cells and HCT15 cells at 2.5:1, 5:1, and 10:1 (E:T) ratios for 4 hours. (**C** and **D**) Expression of CD69 and CD107a in the indicated group was performed by flow cytometry after coculture with HCT116 cells at a 5:1 ratio for 4 hours. The representative FACS histogram (left) and the MFI column chart (right) are displayed. (**E** and **F**) FACS analysis of GzmB and IFN-γ expression in indicated groups after co-culture with HCT116 cells at a 5:1 ratio for 4 hours. The representative FACS profile is shown on the left, and the MFI column chart is on the right. The experiment was performed three times. Data are shown as means ± SD. Analyzed by one-way ANOVA (*P < 0.05, **P < 0.01, ***P < 0.001, and ****P < 0.0001).

According to the methods in our previously published paper (*23*, *26*), we developed an NKG2D/4-1BBζ-NK cells with a knockdown of GPR132 (fig. S17, A and B), which performed considerable GPR132 interference efficiency at RT-PCR and WB levels (fig. S17, C and D). As target cells, colorectal cancer cell lines HCT116 and HCT15 display high expression of NKG2D ligand MICA/B (fig. S18). Therefore, we incubated Mock-NK92 cells, CAR-NK92 cells, NC-CAR-NK92 cells, and SH-CAR-NK92 cells with HCT116 and HCT15 for 4 hours, respectively, with the 2.5:1, 5:1, and 10:1 E:T ratio. The data indicated that NK92 cells expressing CAR could effectively kill target cells, whereas SH-CAR-NK92 cells demonstrated enhanced cytotoxicity (Fig. 7, A and B). To detect the expression of NK activation–related proteins, CAR-NK92 cells were cocultured with HCT116 for 4 hours at a 5:1 (E:T) ratio. The results revealed that SH-CAR-NK92 cells expressed higher levels of CD69 and CD107a than CAR-NK92 cells and NC-CAR-NK92 cells (Fig. 7, C and D). In addition, the expression of GzmB and IFN-γ was higher in SH-CAR-NK92 cells than in the other groups (Fig. 7, E and F). The aforementioned results indicated that GPR132 knockdown increased the cytotoxicity capability of CAR-NK92 cells. Furthermore, it was observed that the killing capacity of SH-CAR-NK92 cells remained stronger than that of NC-CAR-NK92 cells even after a 12-hour treatment with lactate. The cytotoxicity of the lactate-treated NC-CAR-NK92 cells was notably reduced compared to the untreated group, while in lactate-treated SH-CAR-NK92 cells, the reduction was not significant (fig. S19, A and B). Briefly, the down-regulation of GPR132 could enhance the CAR-NK cell function and could attenuate the inhibition of CAR-NK cell function by lactate.

### Knockdown of GPR132 enhances the antitumor activity of CAR-NK92 cells in vivo

**Fig. 8.**
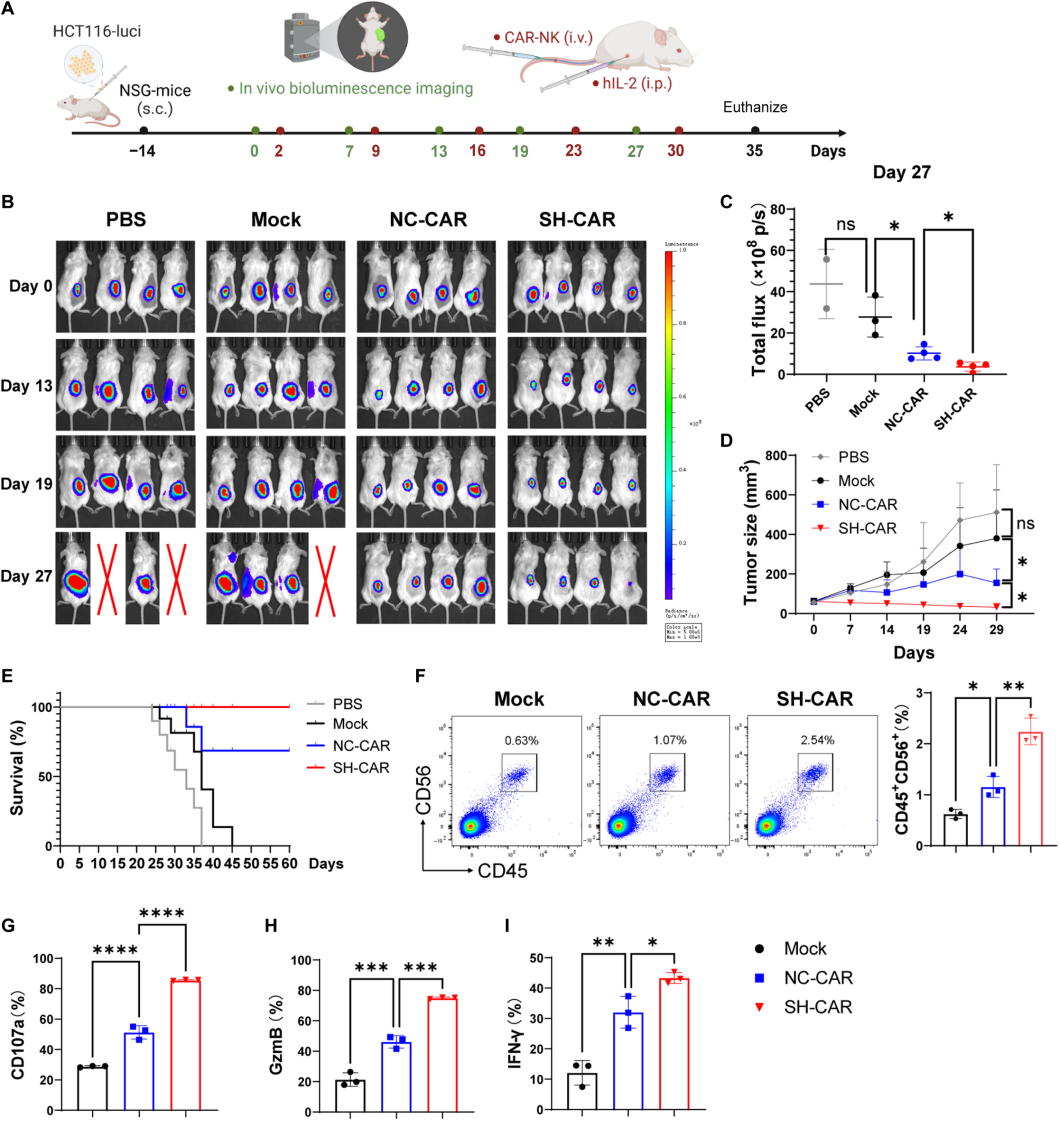
Down-regulation of GPR132 enhances the antitumor ability of CAR-NK92 cells in vivo. (**A**) Schematic representation of the animal experimental design. (**B**) IVIS was used to assess the progression of HCT116-luciferase tumors in each group on days 0, 13, 19, and 27 (*n* = 4). (**C**) On day 27, the total bioluminescence flux (photons per second) emitted by tumors of the remaining mice in each group was quantified through IVIS. (**D**) Tumor volume was regularly assessed using calipers and presented with line plots (*n* = 4). (**E**) Overall survival rate for mice in each experimental group was depicted by the Kaplan-Meier survival curve (*n* = 8). (**F**) FACS analysis of the proportion of NK cells (CD45^+^CD56^+^) inside the tumor (*n* = 3). The representative FACS profile is shown on the left, and the column chart is on the right. (**G** to **I**) Expression of CD107a, GzmB, and IFN-γ in tumor CD45^+^CD56^+^ cells was determined by FACS (n = 3). Data are shown as means ± SD and were analyzed by one-way ANOVA (*P < 0.05, **P < 0.01, ***P < 0.001, and ****P < 0.0001).

Next, we further evaluated the ability of GPR132-knockdown CAR-NK cells to eradicate colorectal cancer in vivo. Considering that HCT116 cells are more sensitive to NKG2D-CAR-NK92 cells than HCT15 cells, HCT116 cells were selected to establish a colorectal cancer xenograft mouse model. A total of 3 × 10^6^ HCT116-luciferase cells were injected subcutaneously into NSG mice. Mice were randomly divided into four groups when tumor volumes reached 60 to 100 mm^3^ and received PBS, Mock-NK92 cells, NC-CAR-NK92 cells, and SH-CAR-NK92 cells (5 × 10^6^ cells per mouse) through the tail vein, respectively. At the same time, hIL-2 (20,000 IU per mouse) was injected intraperitoneally. Weekly injected the treatment NK92 cells and hIL-2 and performed in vivo imaging system (IVIS) and tumor measurements every few days (Fig. 8A). The results showed that the SH-CAR-NK92 cells exhibited a marked antitumor capacity in vivo compared to Mock-NK92 and NC-CAR-NK92 cells, and the volume of tumors from mice treated with SH-CAR-NK92 cells was lower than in the other groups (Fig. 8, B to D, and fig. S20, A and B). In addition, the mice treated with SH-CAR-NK92 cells showed a higher survival rate, as indicated in Fig. 8E. We also evaluated the number and activation markers of CAR-NK92 cells inside the tumor. The results indicated that the proportion of tumor-infiltrating NK cells in the SH-CAR-NK92 group was the highest (Fig. 8F), and the expression of CD107a, GzmB, and IFN-γ was remarkably elevated compared to other groups (Fig. 8, G to I, and fig. S20, C to E). The findings definitely prove that the down-regulation of GPR132 can effectively improve the anti-colorectal cancer efficacy of CAR-NK cells in vivo.

## DISCUSSION

In recent years, cancer immunotherapy has revolutionized the field of oncology, including cytokine therapy, IBD, adoptive cell therapy, cancer vaccines, monoclonal antibodies, and CAR therapy (*27*). Among them, the application of therapeutic monoclonal antibodies (mAbs) to immunosuppressive “checkpoint” receptors has fundamentally rewritten the traditional model of cancer treatment (*28*). Recently, there has been a surge of interest in NK cell adoptive therapies. Unlike T cells, NK cells can recognize and respond to cancerous cells without prior exposure. In addition, allogeneic NK cells do not trigger immune rejection, which strongly promotes the emerging development of NK cell adoptive therapy. The combination of NK adoptive cells and ICB therapy could achieve remarkable tumor control (*29*, *30*). However, the majority of clinical patients tolerate immune checkpoint inhibitors (*31*), and the clinical efficacy of adoptive NK cell therapy in solid tumors is also unsatisfactory. Therefore, it is essential to identify new immune checkpoints and use them to enhance the antitumor immune response of NK cells.

GPCRs are described as very attractive drug targets and play an important role in the regulation of NK cell function. It has been reported that substance P promotes the expression of perforin and GzmB in NK92-MI cells through NK-1R (GPCR) signaling (*32*). Similarly, the activation of GPR55 enhances NK cell activation and IL-12 and TNF-α production (*33*). However, Chang *et al.* (*34*) reported that GPR56 expression is down-regulated in activated NK cells and inhibited cytotoxicity and cytokine production of NK cells. In addition, adenosine receptors are expressed in tumor-infiltrating NK cells and limit the maturation of NK cells, making it a potential immune checkpoint (*35*, *36*). Moreover, previous studies in our laboratory demonstrated that GPR116 negatively regulates NK cell function through the Gαq/HIF1α/NF-κB signaling pathway (*23*). In the present study, RNA-seq results showed that GPR132 was substantially down-regulated in activated NK cells, suggesting that GPR132 may regulate the NK cell function.

The proton-sensitive GPCR family is sensitive to low pH and regulates cell proliferation and metastasis, immune cell function, inflammation, and vascularization (*6*). Parks *et al.* (*8*) found that the deletion of GPR132 promoted the migration of macrophages. In addition, increased transcription of GPR132 leads to B cell differentiation blockage (*9*). These studies showed that GPR132 plays an important role in regulating immune cell function. Therefore, we first performed a preliminary study using GPR132^−/−^ mice. Le *et al.* (*37*) previously reported that the deletion of GPR132 promotes T cell proliferation in aged mice (over 1 year old). Our findings indicated that GPR132^−/−^ mice (6 to 8 weeks old) had a higher proportion of NK cells but with no significant difference in other immune cells. Accordingly, we performed melanoma lung metastasis and bone marrow transplantation experiments, further demonstrating the absence of GPR132 promotes the antitumor ability of NK cells. Subsequently, we further observed the effects of GPR132 on NK cell function using shRNA and ONC212 agonists. The results confirmed that the reduction of GPR132 levels could boost NK92 cell function, while activation did the opposite.

NF-κB serves as a crucial regulator of cytokine and chemokine production in NK cells. In patients with deficiencies in NF-κB components [NEMO, inhibitor of nuclear factor κB (IκB), and IκB kinase β], NK cells display significant impairments in cytotoxic function and IFN-γ production upon recognition of target cells (*38*, *39*). Our KEGG enrichment showed that the NF-κB signaling pathway was significantly enriched in activated NK cells, and the ZAP70 gene is among in it, which is known to play a key role in T cell receptor signaling and regulates the activation of protein kinase Cθ and NF-κB in T cells (*40*). Hideshima *et al.* (*22*) found that immunomodulatory drugs can directly activate ZAP-70, thereby leading to increased expression of GzmB and activity of NK cells. Our study also confirmed that ZAP70 regulates the expression of NF-κB in NK92 cells. It is widely known that cyclic adenosine 3′,5′-monophosphate (cAMP) is directly produced by G proteins. Within the immune system, cAMP inhibits the activity of Lck through the cAMP-dependent protein kinase (PKA)/CSK pathway, which further leads to the inhibition of ZAP70 activation (*41*). Consistent with the above, our results revealed that GPR132 inhibits ZAP70/NF-κB signaling through the Gαs-PKA-CSK pathway, further inhibiting the function of NK cells. This suggests that GPR132 is a potential immune checkpoint that can be targeted to enhance the antitumor response of NK cells.

Now, CAR-T therapy has revolutionized cancer treatment. Although CAR-T cells have shown effectiveness in hematological cancers, they offer limited therapeutic benefits for solid tumors. Moreover, CAR-T cells may cause graft-versus-host disease, cytokine release syndrome, and neurologic toxicities. In contrast, NK cells provide a safer and more advantageous CAR-engineering platform (*42*). Nevertheless, CAR-NK cells still face challenges in solid tumors, including target selection, CAR engineering, and persistence (*43*). In addition, immunosuppressive TME in solid tumors is another obstacle. Transforming growth factor–β is expressed in the majority of TMEs, which inhibits the tumor lytic response and causes the expression of eomesodermin (EOMES) lost (*44*), ultimately contributing to NK cell exhaustion. TME is also hypoxic, which can down-regulate the expression of activating receptors NKG2D and induce the release of HIF1α in tumor-infiltrating NK cells, thereby reducing their antitumor response and IFN-γ production (*17*).

Furthermore, the TME hinders the effectiveness of NK cells by metabolizing large amounts of lactate. Because GPR132 is reported to be sensitive to lactate in the breast cancer microenvironment (*12*), we treated GPR132-deficient NK92 cells and normal NK92 cells with lactate, and the results demonstrated that the GPR132 knockdown can lessen lactate-induced inhibition of NK92 cells. Although the evidence for GPR132 sensed to low pH is still limited and less reliable than other pH-sensing GPCRs, it can be converted to a collaborative pH sensor by installing the missing glutamate residues (*45*). The genetically modified NKG2D/4-1BBζ-T cells previously reported in our laboratory have enhanced anti-pancreatic and prostate cancer capabilities (*46*–*48*). In addition, NKG2D/4-1BBζ-T cells have been reported to exhibit specific cytotoxic activity against colorectal cancer cells (*49*). On the basis of these findings, we down-regulated GPR132 in NKG2D/4-1BBζ-NK92 (CAR-NK92) cells to detect its anti-colorectal cancer activity. Compared to traditional CAR-NK cells, the GPR132-downregulated CAR-NK cells displayed enhanced cytotoxicity against colorectal cancer cells and increased the expression of GzmB and IFN-γ. In the treatment of colorectal cancer xenografted mice, GPR132-downregulated CAR-NK cells exhibited excellent antitumor activity and intratumoral survival. The 4-1BB/CD3ζ combination has been prioritized in our CAR-NK design as it has shown potent signaling and enhanced cytotoxicity in CAR-NK therapy (*23*, *50*) as well as a high clinical application rate (*51*). However, DAP10 and DAP12 have also been extensively studied as endogenous costimulatory molecules in NK cells (*52*, *53*), providing advanced insights for our future exploration.

In summary, our results revealed that the GPR132 receptor regulates NK cell function through Gαs/CSK/ZAP70/NF-κB signaling axis. The deficiency of GPR132 can attenuate the inhibition of NK cells by lactate, consequently increasing the expression of INF-γ and GzmB and effectively boosting the anti-colorectal cancer effect of CAR-NK92 cells. Our study more conclusively proves that GPR132 is a potential immune checkpoint and provides an advanced avenue for NK cell therapy.

## MATERIALS AND METHODS

### Cell preparation and culture

Human natural killer cell (NK92), chronic myeloid leukemia cell (K562), embryonic kidney cell [human embryonic kidney (HEK) 293T], colorectal cancer cell (HCT116 and HCT15), gastric cancer cell (MGC803), mouse melanoma cell (B16-F10), and mouse lymphoma cell (Yac-1) lines were obtained from American Tissue Culture Collection (ATCC, USA). NK92 was grown in αMEM medium (Procell, China) supplemented with 12.5% fetal bovine serum (FBS; ABW, Uruguay), 12.5% horse serum (Gibco, NY), 0.2 mM inositol, 0.1 mM β-mercaptoethanol, 0.02 mM folic acid (Sigma-Aldrich, USA), hIL-2 (100 to 200 IU/ml; PeproTech, USA), and 1% penicillin-streptomycin (P/S). HEK293T was cultured in Dulbecco’s minimum essential medium (Gibco, USA), and K562, HCT116, HCT15, MGC803, Yac-1, and B16-F10 were cultured in RPMI 1640 medium (Gibco, USA). The culture medium was supplemented with 10% FBS and 1% P/S. Cells were grown at 37°C and 5% CO_2_. In addition, HCT116 and HCT15 cells transduced luciferase-lentiviral to express firefly luciferase as the reporter.

### Construction of lentiviral expression plasmid

Human GPR132 mRNA (GenBank: EU431121.1) was obtained from the National Center for Biotechnology Information as a template to design shRNA. Three GPR132-shRNA sequences and a NC sequence were randomly selected from the website of BLOCK-iT RNAi Designer (listed in table S1). At the 3′ end of the shRNA sequence, the loop sequence, respective reverse complementary sequence, 6Ts tail, and EcoR І cleavage site were added sequentially. Subsequently, inverse complementary sequences were generated, and the Xho I sticky and Hpa I sticky were added to the ends of the two complementary nucleic acid strands, respectively. To obtain the interfering plasmids, the synthesized shRNA sequences were annealed and ligated to a pLL3.7 vector containing the U6 promoter and enhanced green fluorescent protein. In the CAR-NK study, the most efficient shRNA sequence and NC sequence were inserted into the pLL3.7-NKG2D-CAR plasmid, which is preserved in our laboratory (*46*).

### Mice

Global knockout mice GPR132^−/−^ was developed with CRISPR technology on a C57BL/6 background (fig. S21) and gifted by W. Lu. Genotyping was performed by PCR using primers (forward 5′-TGGA-CTTCCTCCCCTGATCC-3′; reverse 5′- AAACGCAGGTAGTGGTAGCC-3′), and PCR products were sequenced to identify the genotype. NOD/SCID/IL2Rγ-chain^−/−^ (NSG) mice are NOD background. All mice were fed, and animal experiments were performed at specific pathogen–free conditions. The animal experiments protocols were approved by the Institutional Animal Ethics Committee of East China Normal University (m20230903).

### Bone marrow transplant

First, WT and GPR132^−/−^ femurs were harvested and stored in solution A (RPMI 1640 + 2% FBS + 1% P/S). Second, the femoral bone marrow cavity was flushed with a 1-ml syringe, and the cell suspension was filtered with a sterilized cell filter (40 μm). Then, the cells were washed three times with B solution (RPMI 1640 + 1% P/S). The cells were resuspended and counted after centrifuging at 4°C, 300*g*, for 5 min. Last, 5 × 10^6^ bone marrow cells from WT or GPR132^−/−^ mice were injected intravenously through the tail vein into WT recipient mice, which were irradiated with lethal doses (9.5 gray of γ-ray). One month later, the proportion of NK cells in the peripheral blood of recipient mice is analyzed. Moreover, 5 × 10^5^ B16-F10 cells were injected subcutaneously into recipient mice for further study. A summary diagram is shown in fig. S5A.

### Lung metastasis model

B16-F10 melanoma cell line was used to construct a lung metastasis model in WT and GPR132^−/−^ mice. On day 0, each mouse was injected with 3 × 105 B16-F10 cells via the tail vein. For the depletion of NK cells in mice, 200 μg of anti-NK1.1 antibody (108760, BioLegend, USA) was diluted with phosphate-buffered saline (PBS) and injected intraperitoneally, a total injection volume is 200 μl. The control WT and GPR132^−/−^ mice were injected with PBS. On day 14, the mice were euthanized, the lungs were weighed, and the number of colonies of melanoma lung metastases was counted.

### NK cell isolation

PBMCs were separated from healthy donor blood, which was provided by the Shanghai Blood Center (Shanghai, China). Human primary NK cells were enriched from PBMCs by NK cell isolation kit (L00903, GenScript, China). Mouse NK cells were isolated from fresh WT or GPR132^−/−^ mice spleen with an NK cell isolation kit (130-115-818, Miltenyi, Germany). The cell population was meticulously enriched for NK cells, achieving a purity greater than 99%.

### RNA sequencing

Human NK cells isolated from the PBMCs were divided into two groups (control and treatment) and incubated in a 24-well plate; the treatment group was stimulated by PMA (20 ng/ml) and ionomycin (1 μg/ml). After 6 hours, cells were collected using TRIzol reagent (Megan, China) and flash freezing by liquid nitrogen. RNAs were then extracted subsequently, and its integrity was evaluated using the Agilent 2100 Bioanalyzer (Agilent, USA). The libraries were constructed using the Fast RNA-seq Lib Prep Kit V2 (RK20306, ABclonal). The libraries were then sequenced using an Illumina NovaSeq 6000 (PE150) platform and generated approximately 40 million paired-end reads (Novogene, China). The raw read counts were extracted and then normalized by DESeq2. R-pheatmap generated an expression heatmap according to cluster analysis of the gene FPKM value. The Gene Ontology and KEGG pathway enrichment analysis of differentially expressed genes was performed with P-clusterProfiler (V3.0.3).

### Reverse transcription PCR

Total RNAs were extracted from NK92 cells and subjected to reverse transcription for cDNA synthesis. The specific primer sequences used for GPR132, Gαs, Gαi, and β-actin are listed in table S2. The RT-PCR procedure included an initial denaturation step at 95°C for 5 min, followed by 35 cycles consisting of denaturation at 95°C for 30 s, annealing at 60°C for 30 s, and extension at 72°C for 30 s. After completing the cycles, the PCR product was loaded onto a 2% agarose gel for electrophoresis.

### Western blot

Following the procedures outlined in our previously published papers (*23*, *26*), we collected cells and lysed them using radioimmunoprecipitation assay buffer (Epizyme, China), which was supplemented with protease and phosphatase inhibitors. We measured the protein concentration with a BCA protein assay kit (Biosharp, China). The proteins were then separated on 10% SDS–polyacrylamide gels and transferred onto polyvinylidene difluoride membranes (Millipore, USA). After a transfer time of 2 hours, the membranes were blocked with 5% skim milk and incubated overnight at 4°C with primary antibodies. Following this, the membranes were washed three times with TBST and subsequently incubated for 30 min with an horseradish peroxidase–labeled goat anti-rabbit antibody (Epizyme, China). Last, the immunoreactive signals were detected using the ECL detection system (Epizyme, China). The primary antibodies used were rabbit anti-GPR132 (DF4894, Affinity, China) and rabbit anti–β-tubulin (LF204, Epizyme, China).

### Single-cell suspension acquisition of mouse tissues

Peripheral blood leukocytes were sampled through the orbital vein, followed by erythrocyte lysis, and filtered with a 40-μm filter (Corning, USA). The mouse femur and tibia were removed muscle, rinsed with a 1 ml syringe of tissue, and followed red blood cell lysis to obtain bone marrow cells. For lung and tumor, the tissues were digested using a cocktail of collagenase type I (0.1 mg/ml; Sigma-Aldrich, USA) and collagenase type IV (1 mg/ml; Sigma-Aldrich, USA), and deoxyribonuclease I (3 mg/ml; Sigma-Aldrich, USA) at 37°C for 30 min; after, cells were filtered through a 40-μm filter to obtain a single-cell suspension. For other tissues, after stripping from mice and triturating, red blood cells were lysis, and filtration was performed to obtain a single-cell suspension.

### Cytotoxicity assays

Annexin V apoptosis assay for the cytotoxicity of effector cells: 3 × 10^4^ target cells (Yac-1, K562) were labeled with carboxyfluorescein diacetate succinimidyl ester (CFSE) dye and seeded in a 96-well plate. Effector cells were incubated at various E:T ratios (mouse splenic NK cells: 5:1, 10:1, 20:1; human NK92 cells: 2.5:1, 5:1, 10:1). After 4 hours of co-incubation, cells were collected and stained with the annexin V antibody, and the positive frequency of annexin V under the CFSE gate was analyzed by flow cytometry. The killing ability of effector cells was quantified after normalizing based on the self-apoptosis of target cells.

Luciferase assay for the cytotoxicity of effector cells: 3 × 10^4^ luciferase-transduced target cells (HCT116, HCT15, and MGC803) were seeded in a low-attachment 96-well plate. Effector cells were incubated at various E:T ratios (2.5:1, 5:1, and 10:1). After 4 hours of co-incubation, cells were collected, and the killing ability of effector cells was detected using the Firefly Luciferase Reporter Gene Assay Kit (Beyotime Biotech, Shanghai, China). Alternatively, the cytotoxicity was analyzed by Living Image software (version 4.4) after photographing with bioluminescence imaging.

### Flow cytometry analysis

Cells were washed with PBS supplemented with 2% FBS and 2 mM EDTA [fluorescence-activated cell sorting (FACS) buffer]. To stain the surface markers, cells were incubated with FACS antibodies for 30 min. For intracellular staining of GzmB, IFN-γ, perforin, CSK, ZAP70, and NF-κB, cells were fixed and permeabilized using the BD Cytofix/Cytoperm Kit (BD Biosciences, USA), according to the instructions. A list of the antibodies used is provided in table S3. Data were acquired from either the BD LSRFortessa or the Canto II flow cytometer and were analyzed by FlowJo software (version 10.8.1).

### Mouse models

To establish a subcutaneous melanoma tumor model, 5 × 10^5^ B16-F10 cells were subcutaneously injected into the right back flank of WT or GPR132^−/−^ mice aged 6 to 8 weeks. The tumor volume was regularly measured using calipers, applying the formula *V* = 1/2 (length × width^2^). When the tumor size reached 1500 mm^3^, the mice were euthanized, and tumor tissues were weighted and collected for the preparation of single-cell suspension.

To evaluate the therapeutic effect of CAR-NK in vivo, 8-week-old female NOD/SCID/γ-chain^−/−^ (NSG) mice were used to establish a colorectal cancer xenograft model. A total of 3 × 10^6^ HCT116-luciferase cells were subcutaneously injected into the right back flank of NSG mice. When tumor volume reached approximately 60 to 100 mm^3^, the mice were randomly divided into four groups (*n* = 8) and accessed with an IVIS. Mice were administered PBS or Mock-NK92, CAR-NK92, and SH-CAR-NK92 cells intravenously every 7 days, for a total of five treatments. During the treatment, 20,000 IU of hIL-2 was injected intraperitoneally. Tumor volume was regularly assessed with calipers and calculated using the formula *V* = 1/2(length × width^2^). Alternatively, tumor size was quantified with total bioluminescence flux (photons per second) emitted from the tumors using IVIS. On day 35, the mice were euthanized, and tumor tissues were collected for detection of the proportion and activity of CAR-NK.

### Statistical analysis

GraphPad Prism (version 9.0.0) was used to create the graphs and perform the statistical analyses. All data are expressed as the means ± SD. The differences between the two groups were assessed using an unpaired *t* test. One-way analysis of variance (ANOVA) was used to compare differences among three or more groups, while two-way ANOVA was applied when analyzing two or more variable factors. *P* value of ≤0.05 was considered statistically significant, with the following significance levels: *P < 0.05, **P < 0.01, ***P < 0.001, and ****P < 0.0001. *P* value labeled as “ns” indicates not significant.

## Supplementary Materials

### This PDF file includes:

Figs. S1 to S21 Tables S1 to S3 Original FACS data Original gel images
