# Supplementary material for: GPR132 regulates the function of NK cells through the Gαs/CSK/ZAP70/NF-κB signaling pathway as a potential immune checkpoint
Source: Sci Adv. 2025 Mar 5;11(10):eadr9395. doi: 10.1126/sciadv.adr9395 (PMC11881902; doi:10.1126/sciadv.adr9395)
Supplement: Supplementary file 1 — Figs. S1 to S21 Tables S1 to S3 Original FACS data Original gel images [file sciadv.adr9395_sm.pdf]

Science Advances
AAAS

Supplementary Materials for

GPR132 regulates the function of NK cells through the Gαs/CSK/ZAP70/NF-κB signaling pathway as a potential immune checkpoint

Wenzheng Jiang *et al.*

Corresponding author: Wenzheng Jiang, wzjiang@bio.ecnu.edu.cn

Sci. Adv. **11**, eadr9395 (2025)
DOI: 10.1126/sciadv.adr9395

This PDF file includes:

Figs. S1 to S21
Tables S1 to S3
Original FACS data
Original gel images

Fig. S1.

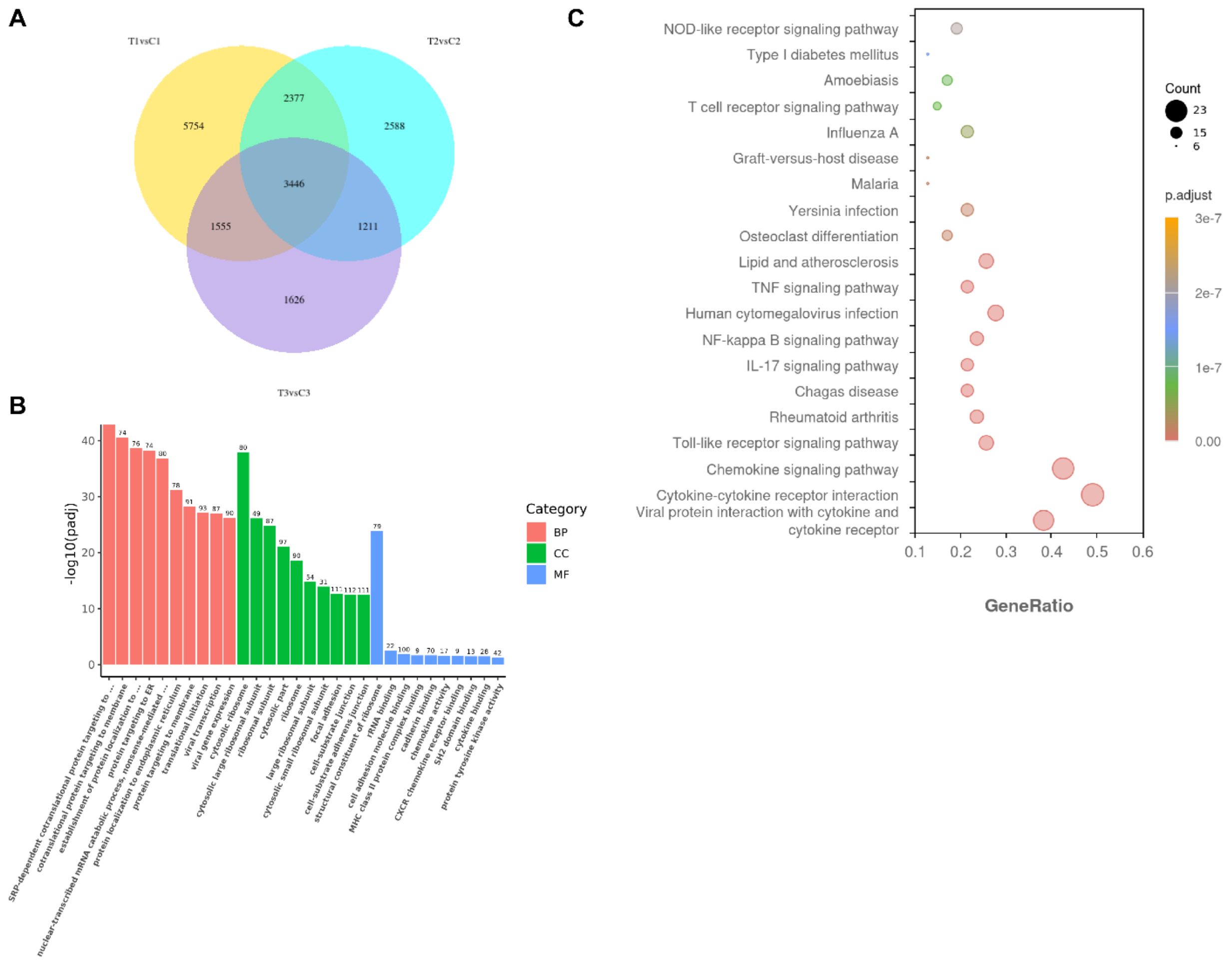

Fig. S1. Differential gene enrichment in activated and inactivated human NK cells. (A) The Venn diagram shows the differential genes shared by 3 donors. (**B)** GO-pathway enrichment of 3446 differential genes shared by 3 donors. BP, Biological Process; CC, Cellular Component; MF, Molecular Function. **(C)** Functional genes of NK cells were analyzed using KEGG enrichment.

Fig. S2.

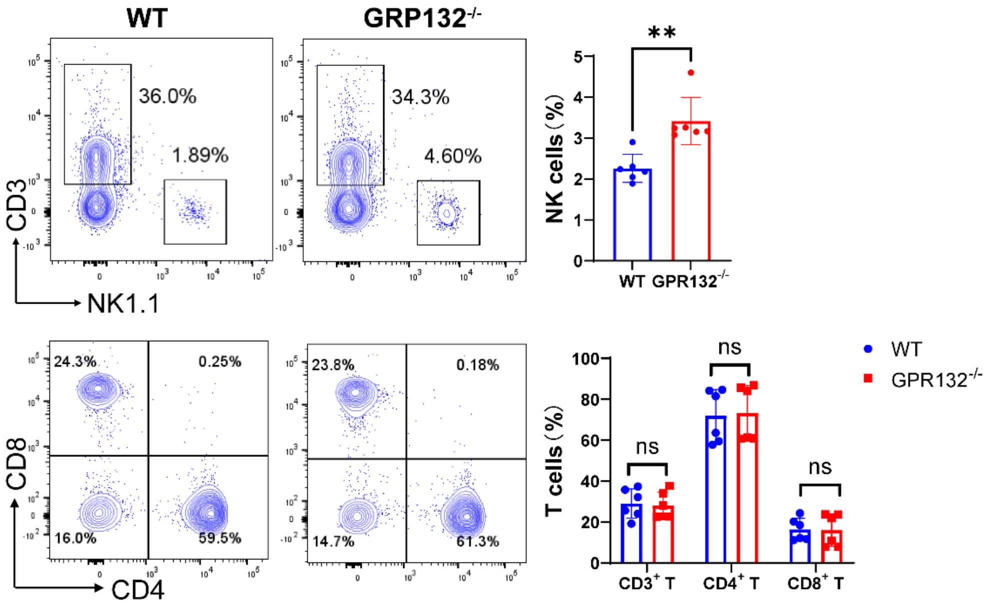

Fig. S2. The proportion of NK cells in the peripheral blood of GPR132$^{-/-}$ mice increased. FACS analysis of NK cell and T cell proportion in peripheral blood of WT and GPR132$^{-/-}$ mice (n=6). The representative FACS profiles are shown on the left, and the column charts are on the right. Data are shown as Mean ± SD and analyzed by unpaired T-tests or two-way ANOVA. (**P < 0.01, ns, not significant)

Fig. S3.

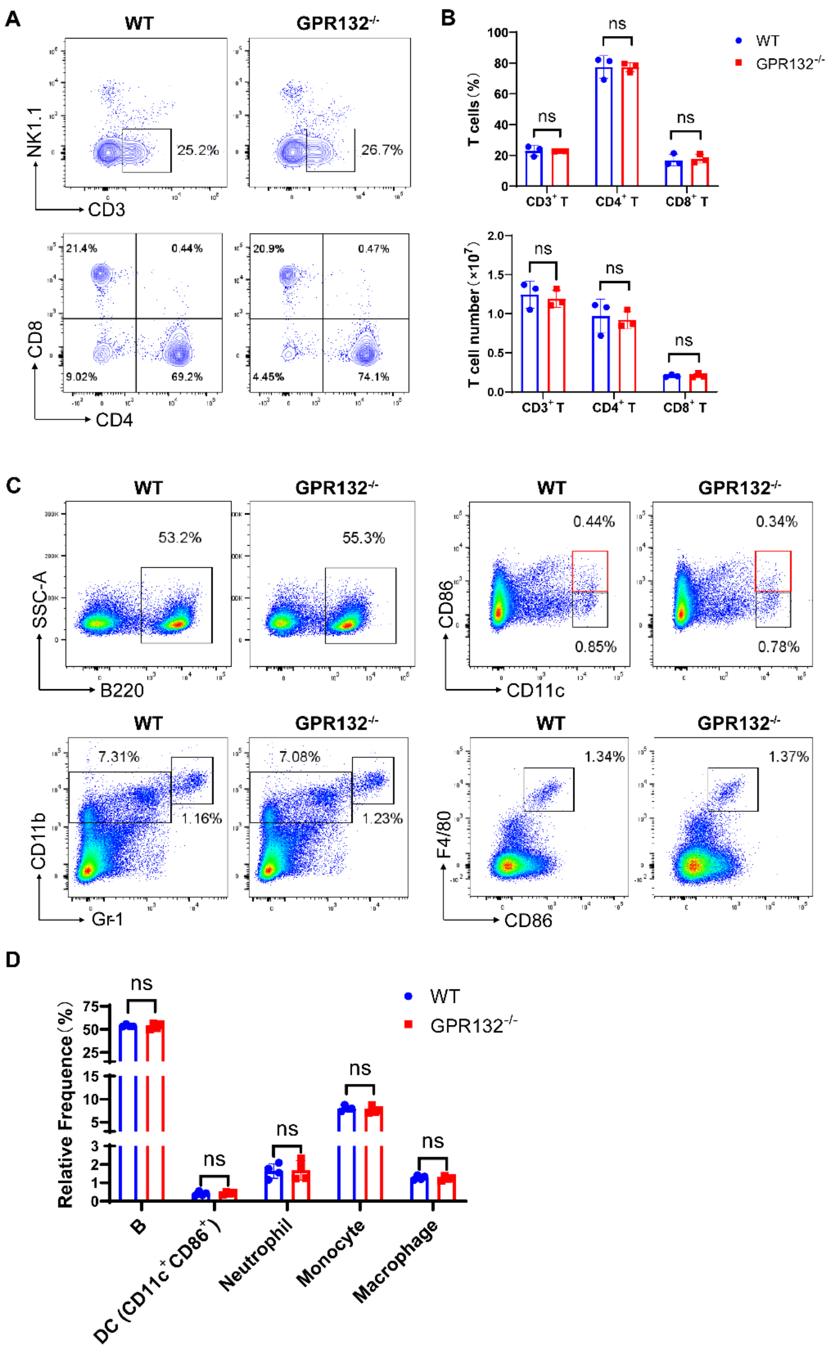

Fig. S3. The proportion of other immune cells in the spleen of GPR132$^{-/-}$ mice shows no significant change. FACS analysis of T cells (**A-B**) and other immune cell proportions **(C-D)** in the spleen of WT and GPR132$^{-/-}$ mice (n=3). Data are shown as mean ± SD and were analyzed by two-way ANOVA. (ns, not significant)

Fig. S4.

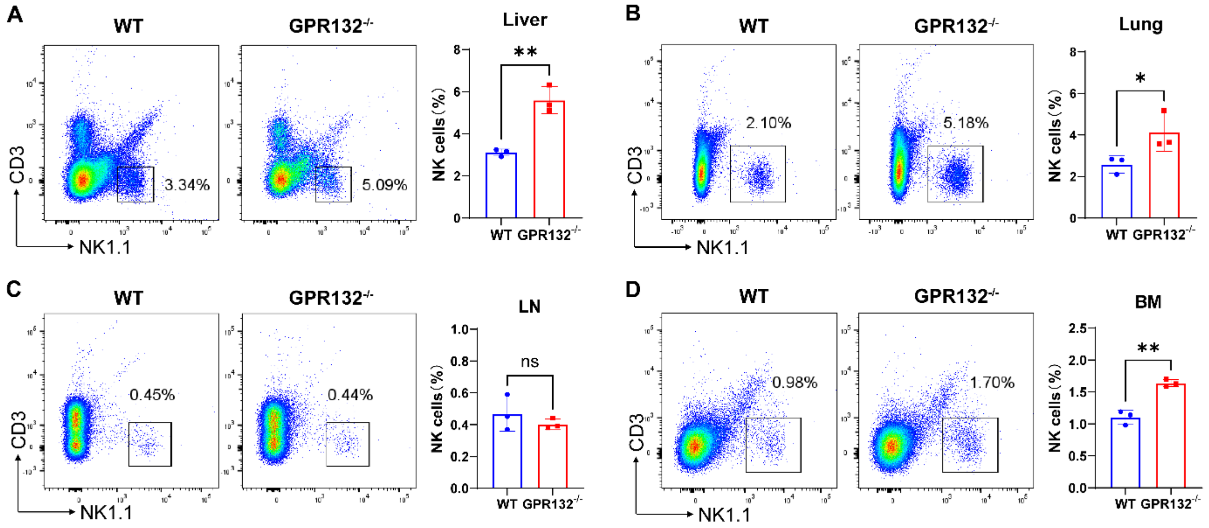

Fig. S4. The proportion of NK cells in the liver, lung, and bone marrow of GPR132$^{-/-}$ mice increased. Data are shown as mean ± SD and were analyzed by unpaired T-test. (*P < 0.05, **P < 0.01, and ns, not significant)

Fig. S5.

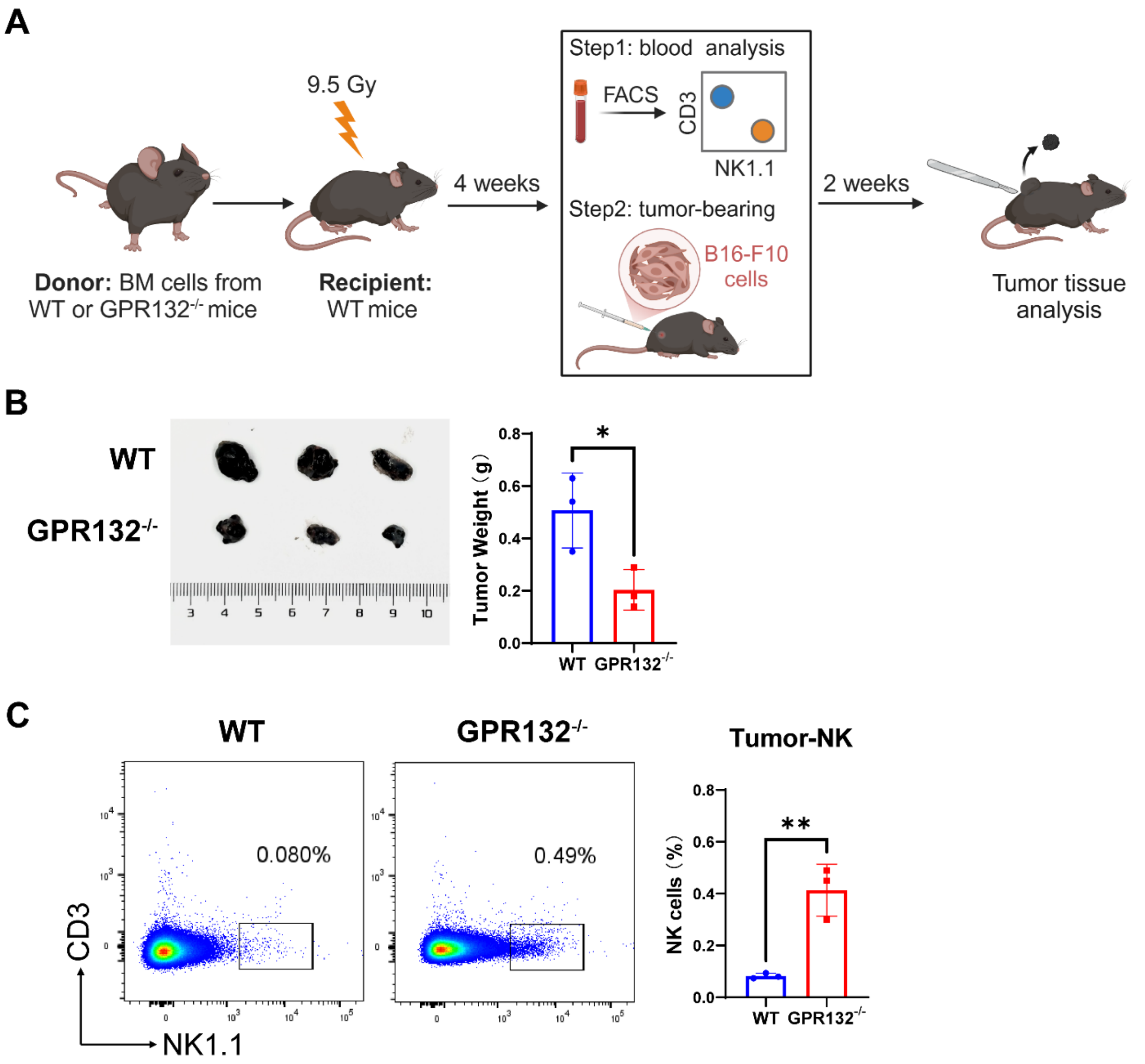

Fig. S5. Bone marrow transplant experiments show that GPR132 inhibited NK cells homeostatically. (A) Schematic representation of the animal experimental design. **(B-C)** Melanomas were divested after tumor-bearing for 2 weeks, photographed, and weighed **(B)**, and then the NK cell proportion inside the tumors was analyzed by FACS (n=3)**. B-C** are shown as Mean ± SD and were analyzed by unpaired T-test. (*P < 0.05, **P < 0.01)

Fig. S6.

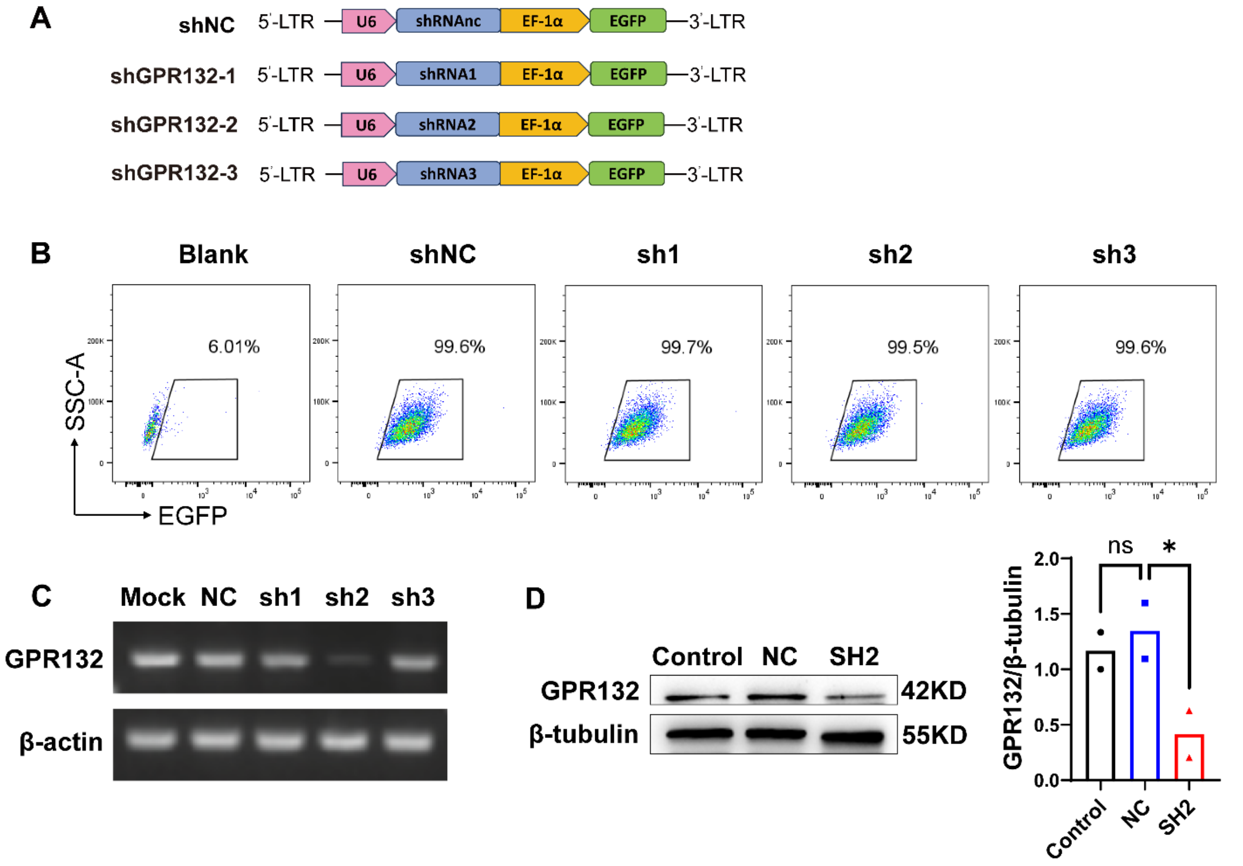

Fig. S6. Screening of GPR132-shRNA. (A) Schematic shows the elements of shRNA-NC, shRNA1, shRNA2, and shRNA3 of GPR132 ORF. **(B)** FACS analysis of the positive NK92 cells (GFP^+) rate after infection with shRNA lentivirus for 48 hours. **(C)** DNA gel electrophoresis displayed the GPR132 expression in NK92 cells after infection with Mock, shRNANC, and GPR132 shRNA1-3 lentivirus. β-actin was used as a reference housekeeping gene. **(D)** Western blot was performed to detect the interference efficiency of shRNA2 with GPR132 protein, and β-tubulin was used as a reference housekeeping gene. (*P < 0.05, and ns, not significant)

Fig. S7.

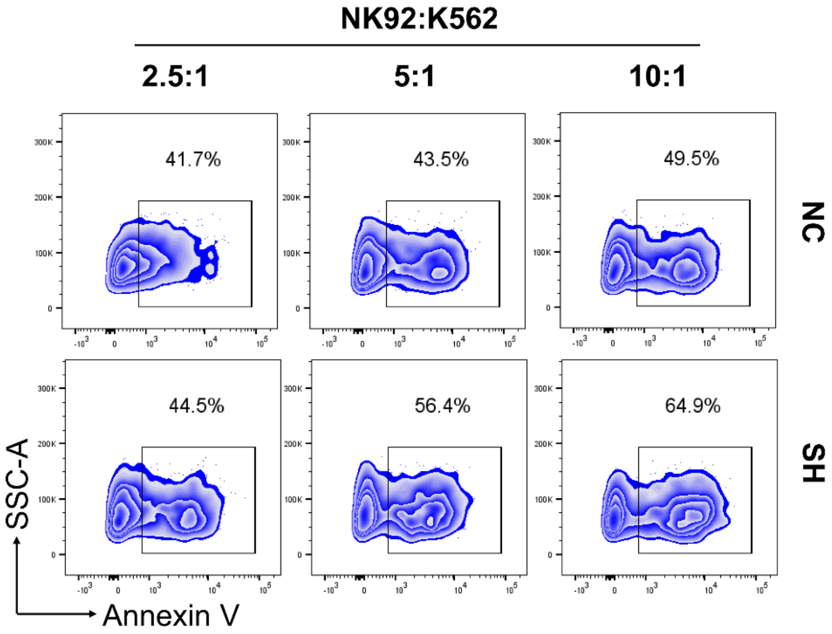

Fig. S7. GPR132-deficient NK92 cells exhibit stronger killing efficiency. The representative FACS profiles show the killing efficiency of NC-NK92 and SH-NK92 cells against K562 cells.

Fig. S8.

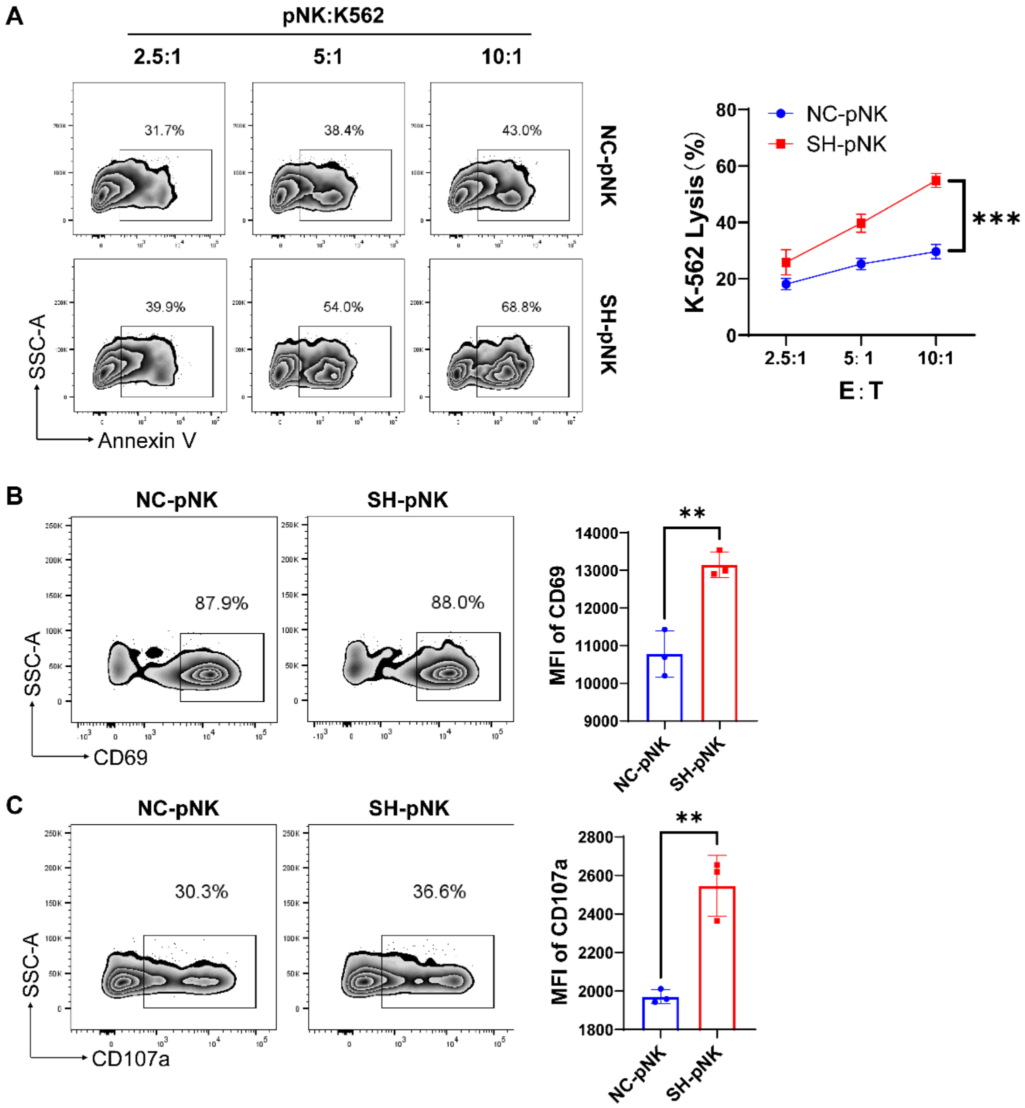

Fig. S8. GPR132-deficient human primary NK cells exhibit stronger cytotoxicity. (A) Primary NK cells were infected with shNC and shGPR132 for 48 h and then co-incubated with K562 cells for 4h at different E: T ratios. **(B-C)** FACS analysis of CD69 and CD107a expression after co-incubated with K562 cells at 5:1 (E: T) ratio. **A-C** are shown as Mean ± SD and were analyzed by unpaired T-test. (**P < 0.01, ***P < 0.001)

Fig. S9.

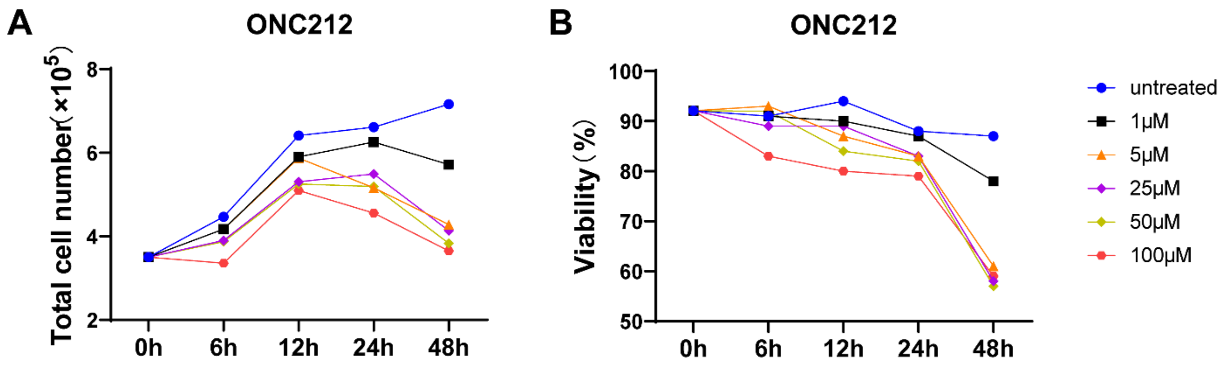

Fig. S9. Exploration of the ONC212 concentration and time-point for treating NK92 cells. To determine the optimal usage conditions for ONC212, wide-type NK92 cells were exposed to various concentrations of ONC212, and cell number and viability were measured at 0, 6, 12, 24, and 48 hours. Viable cell number (**A**) and Viable ratio (**B**)were analyzed by Countstar Mira BF.

Fig. S10.

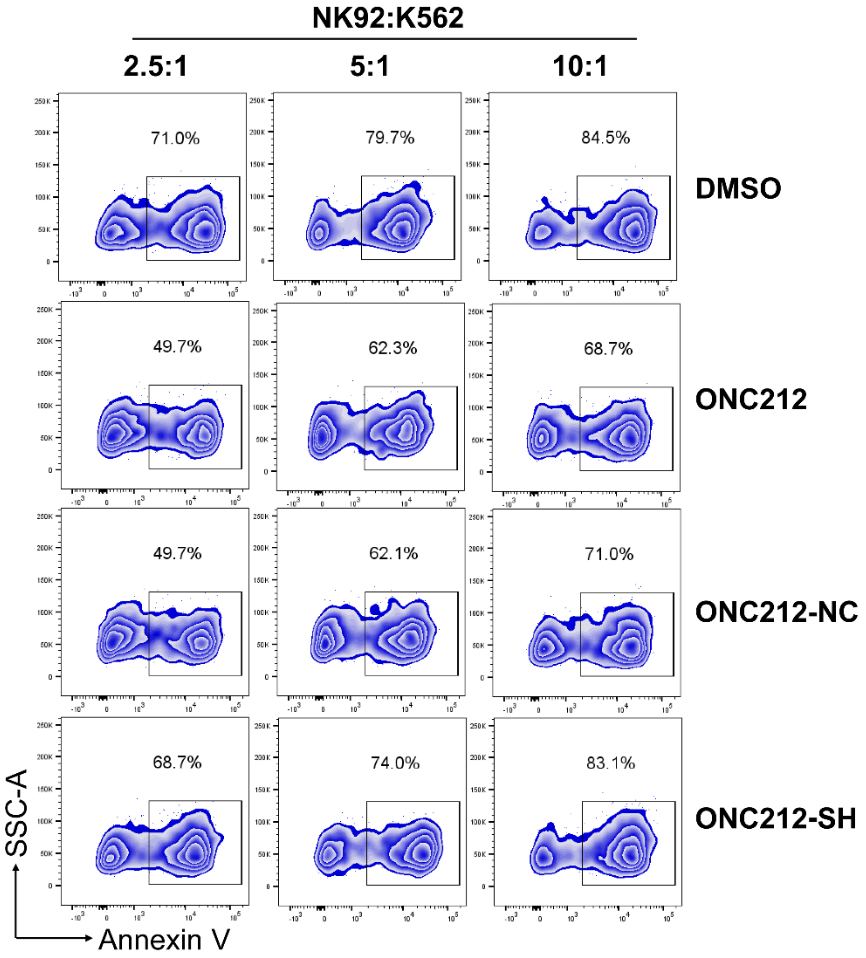

Fig. S10. Activation of GPR132 inhibits the killing efficiency of NK92 cells. The representative FACS profiles show the killing efficiency of NK92 cells, NC-NK92 cells, and SH-NK92 cells, which were treated with DMSO or 5μM ONC212 for 12 hours and co-cultured with K562 cells at different E: T ratios for 4 hours.

Fig. S11.

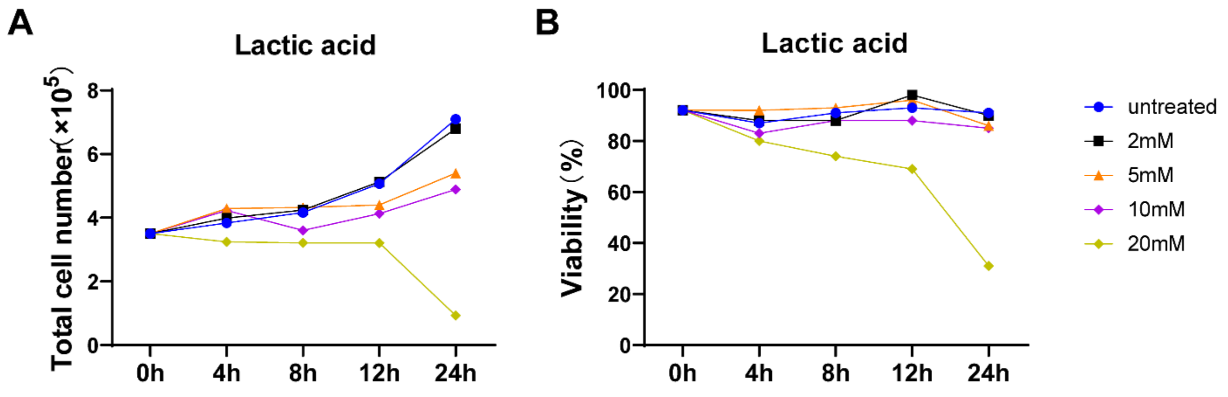

Fig. S11. Exploration of the lactic acid concentration and time-point for treating NK92 cells. To determine the optimal usage conditions for lactate, wide-type NK92 cells were exposed to various concentrations of lactate, and cell number and viability were measured at 0, 4, 8,12, and 24 hours. Viable cell number (**A**) and Viable ratio (**B**) were analyzed by Countstar Mira BF.

Fig. S12.

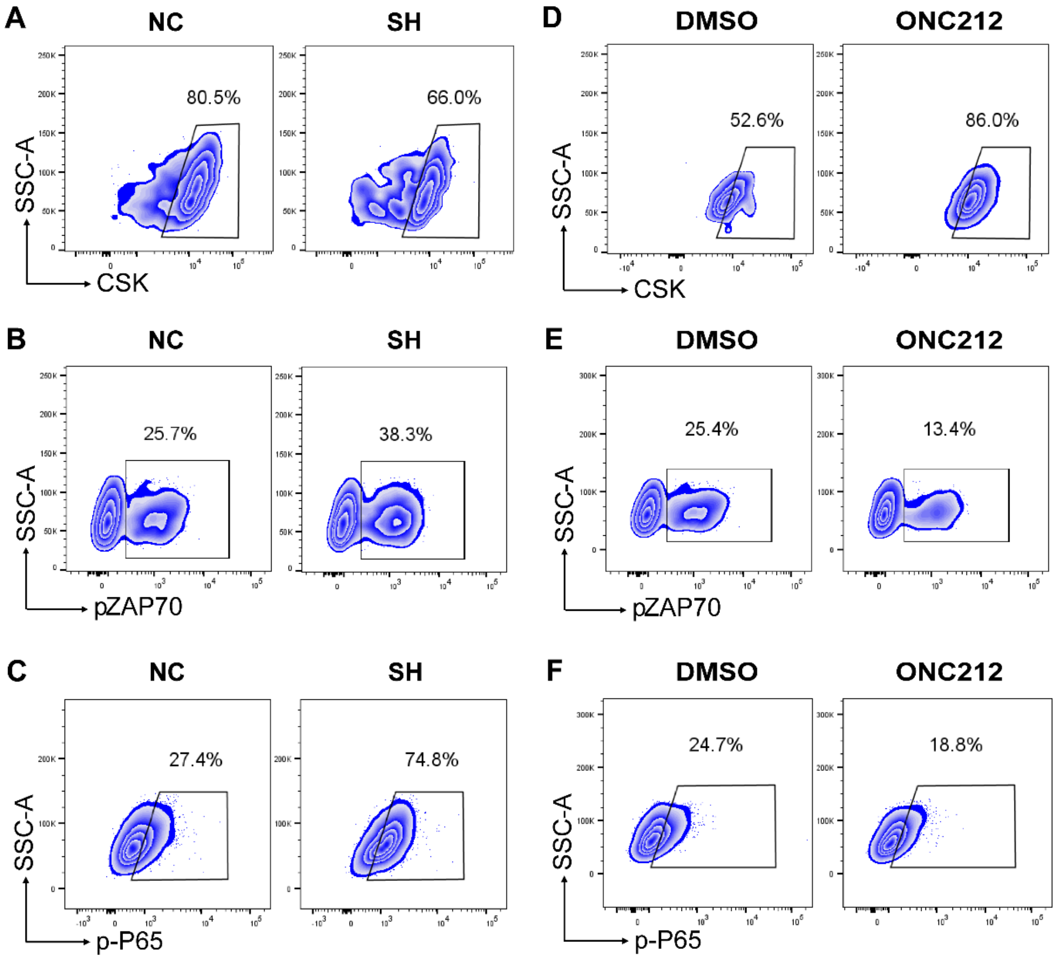

Fig. S12. GPR132 positively regulated the expression of CSK but negatively regulated pZAP70 and p-P65 expression. The representative FACS profiles showed the expression of CSK, pZAP70, and p-P65 in NK92 cells after downregulation **(A-C)** or activation **(D-F)** of GPR132, which corresponds to the column chart of **Figure 6A-F**.

Fig. S13.

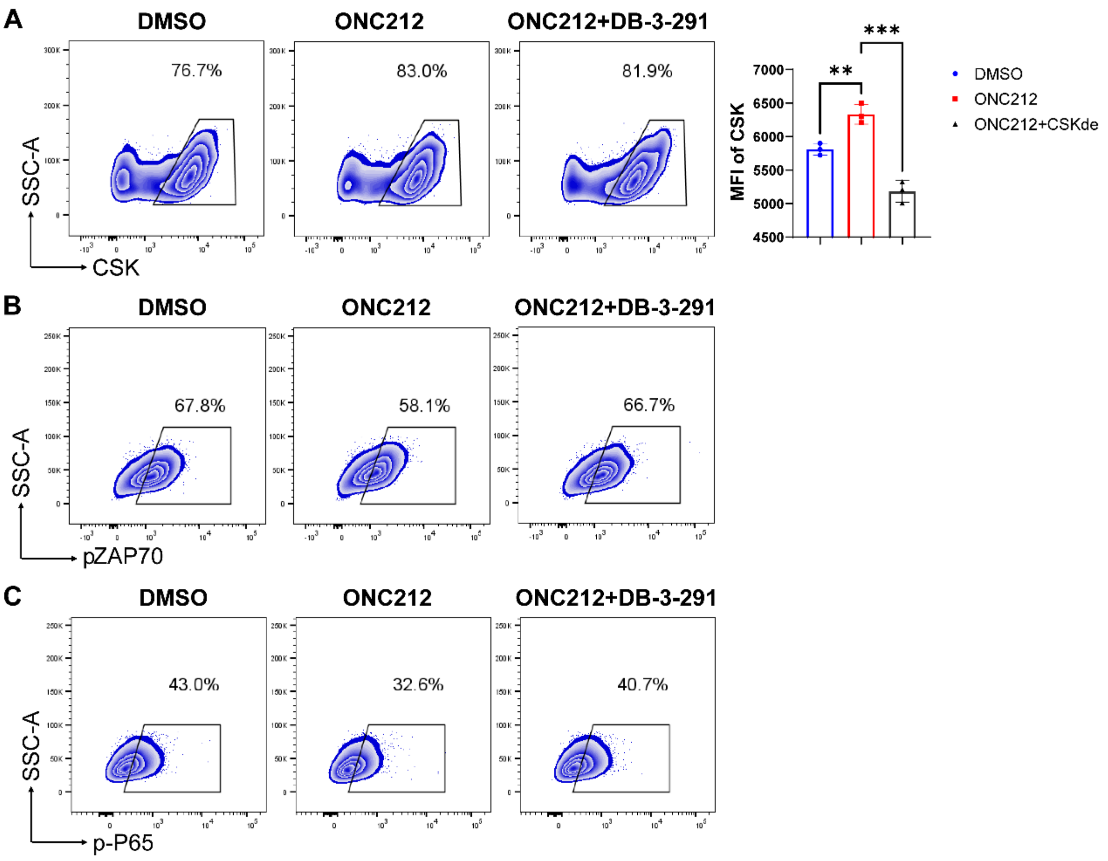

Fig. S13. GPR132 inhibits the expression of pZAP70 and p-P65 via CSK. NK92 cells were treated with DMSO or 10μM DB-3-29 (CSK's degrader) to determine the expression of CSK **(A)**, pZAP70 **(B)**, and p-P65 **(C)** by flow cytometry. For **A**, column data are shown as mean ± SD and were analyzed by one-way ANOVA; For **B-C**, the representative FACS profile corresponds to the column chart of **Figure 6G**. (**P < 0.01, ***P < 0.001)

Fig. S14.

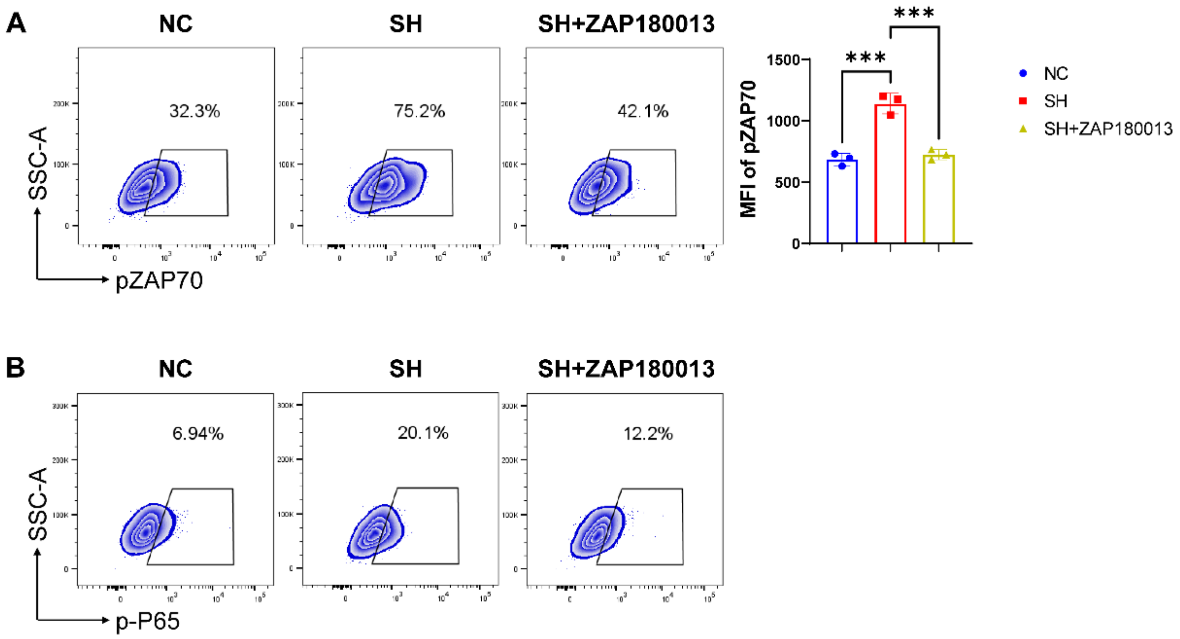

Fig. S14. GPR132 regulates the expression of p-P65 through ZAP70. The expression of ZAP70 **(A)** and p-P65 **(B)** in NC-NK92 and SH-NK92 cells after treatment with DMSO or 5μM ZAP180013 (ZAP70 inhibitor). For **A**, column data are shown as mean ± SD and were analyzed by one-way ANOVA; For **B**, the representative FACS profile corresponds to the column chart of **Figure 6H**. (***P < 0.001)

Fig. S15.

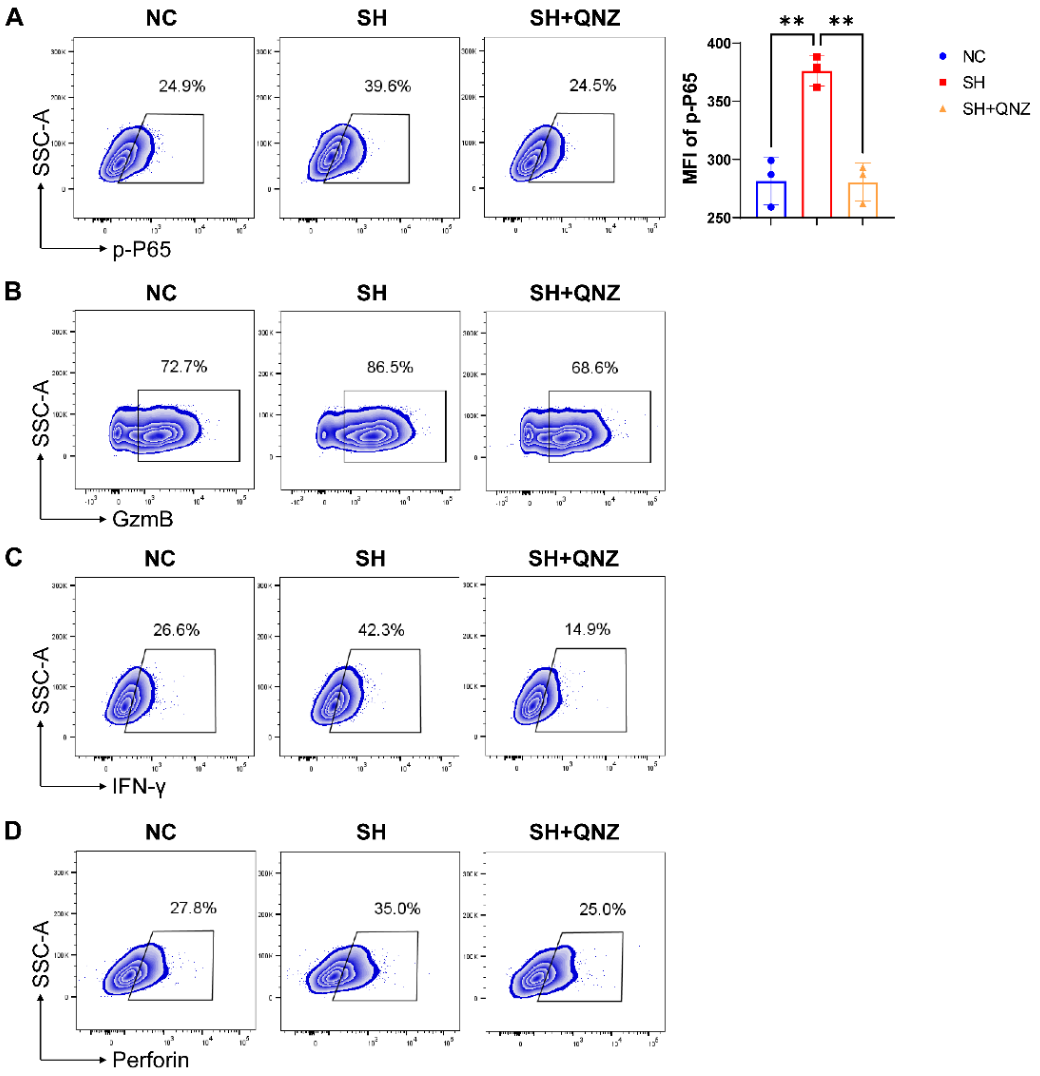

Fig. S15. GPR132 regulates NK cell function through the NF-κB pathway. The expression of p-P65 (A), GzmB (B), IFN-γ(C), and Perforin (D) in NC-NK92 and SH-NK92 cells after treatment with 5μM QNZ (NF-κB inhibitor). For **A**, column data are shown as mean ± SD and were analyzed by one-way ANOVA; For **B-D**, the representative FACS profile corresponds to the column chart of **Figures 6I, J,** and **K**, respectively. (**P < 0.01)

Fig. S16.

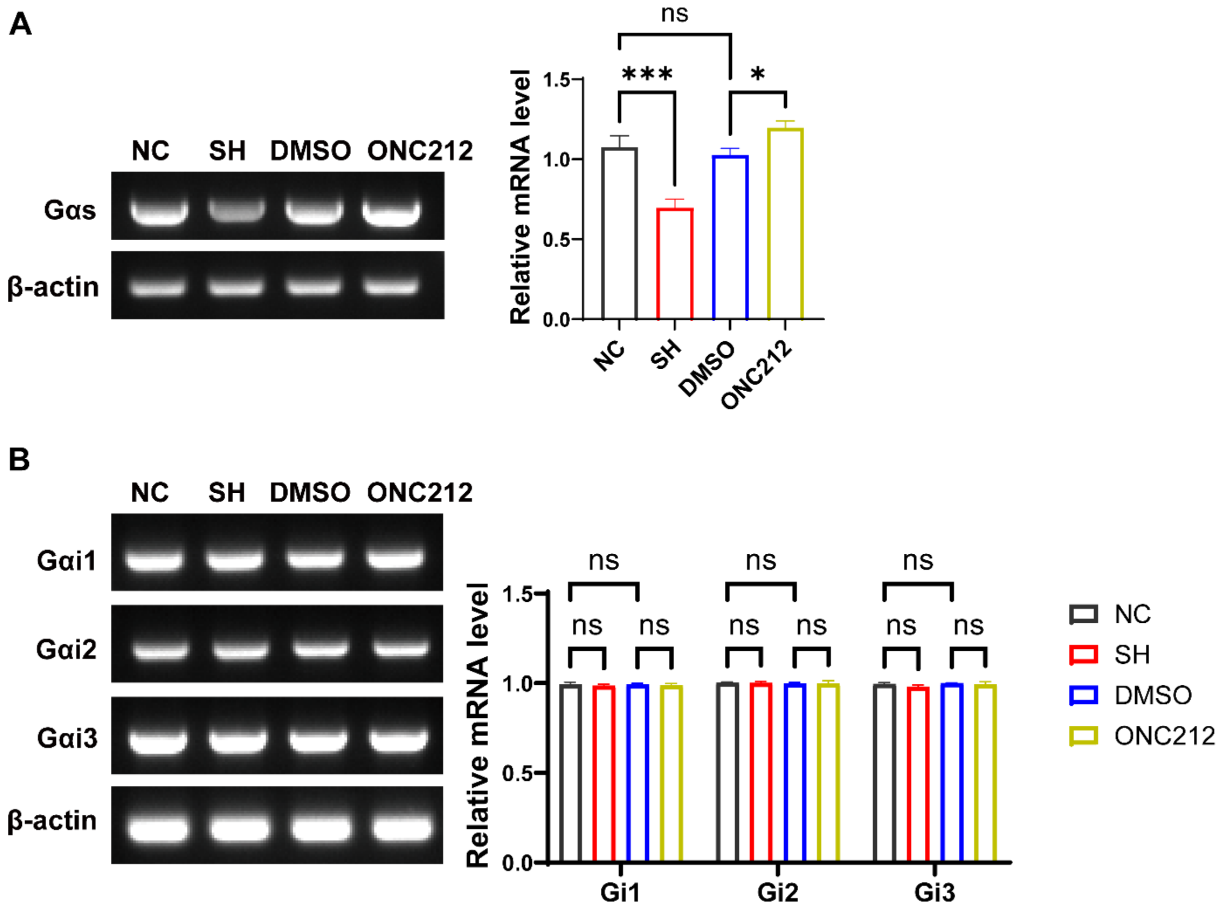

Fig. S16. GPR132 regulates the downstream signaling pathway through Gαs. RT-PCR was used to determine the Gαs and Gαi expression. After infection with the shNC and shGPR132 lentivirus 48 hours, or treatment with DMSO or ONC212 (5μM) 24 hours, NK92 cells were collected and extracted RNA for determination of Gαs (**A**) and Gαi1/2/3 (**B**) expression. Data are shown as mean ± SD. **A** was analyzed by one-way ANOVA and **B** was analyzed by two-way ANOVA. (*P < 0.05, ***P < 0.001, ns, not significant)

Fig. S17.

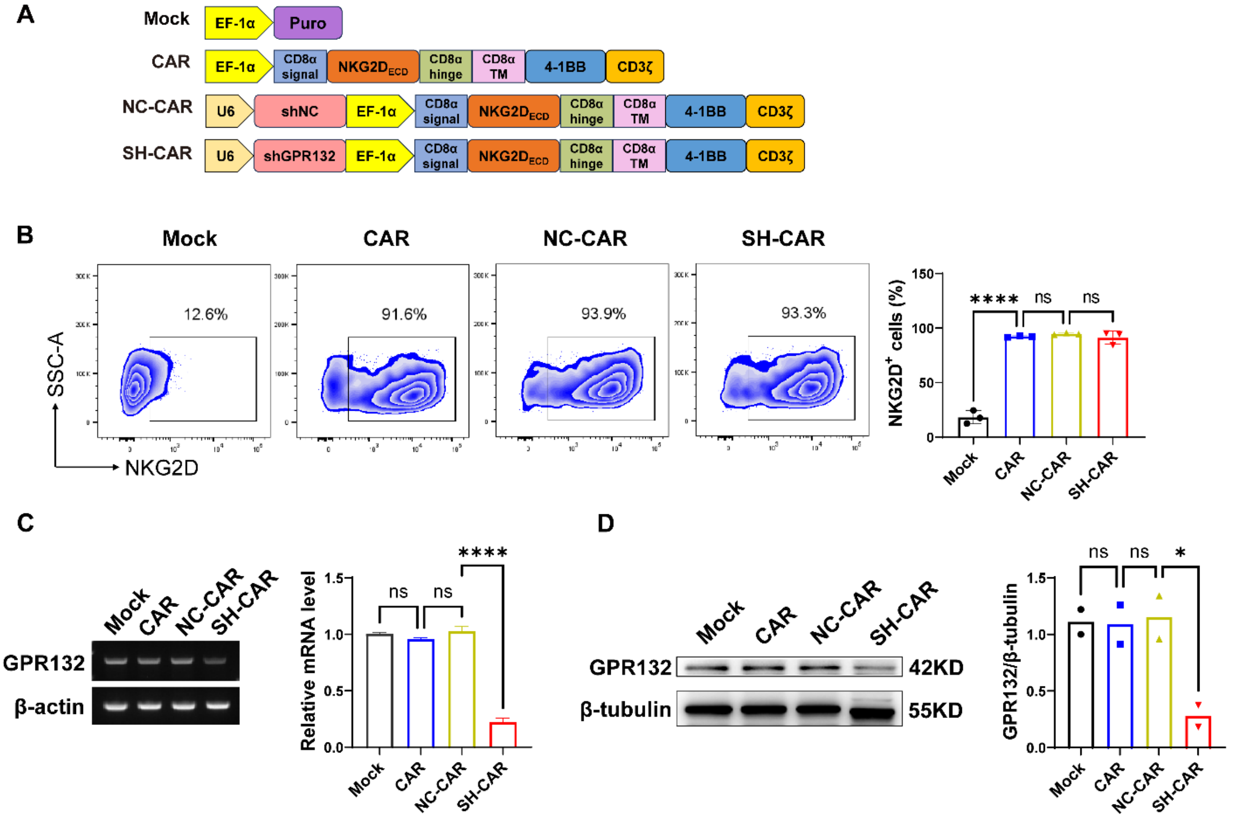

Fig. S17. The generation of SH-CAR-NK92 cells and identification of GPR132 knockdown efficiency. (A) Elements schematic of CAR ORF. Mock is a negative control, and CAR and NC-CAR are non-interfere with GPR132, which is distinguished from SH-CAR. **(B)** FACS analysis of CAR efficiency in CAR-NK92 cells being infected with lentivirus for 48 hours. The representative zebra profile is shown on the left, and the column bar is on the right. **(C)** RT-PCR displayed the GPR132 expression in NK92 cells after infection with Mock, CAR, NC-CAR, and SH-CAR lentivirus. β-actin was used as a reference housekeeping gene. **(D)** Western blot was employed to detect the expression of GPR132 protein in SH-CAR-NK92 cells. Data are shown as mean ± SD and were analyzed by one-way ANOVA. (*P < 0.05, ****P < 0.0001, ns, not significant)

Fig. S18.

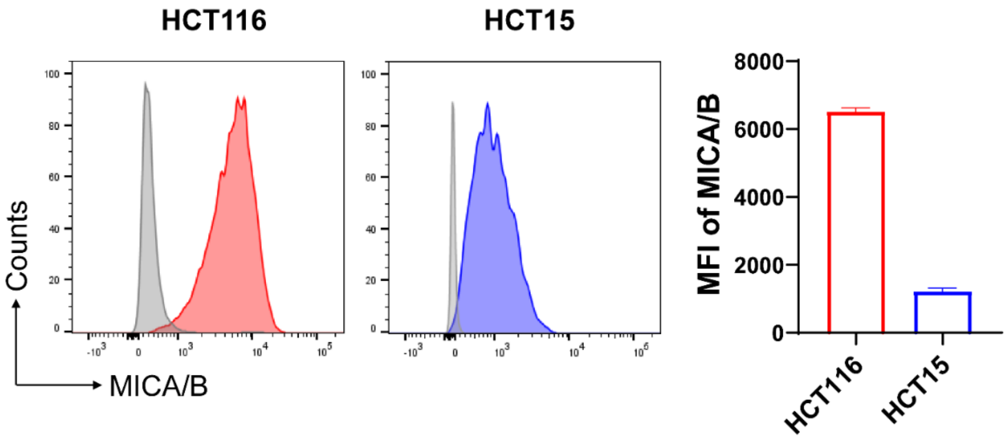

Fig. S18. Colorectal cancer cell lines express considerable levels of NKG2D ligand. FACS analysis of the MICA/B (NKG2D ligand) expression in HCT116 and HCT15 cancer cell lines. The representative FACS histogram is shown on the right, and the MFI column chart is on the left.

Fig. S19.

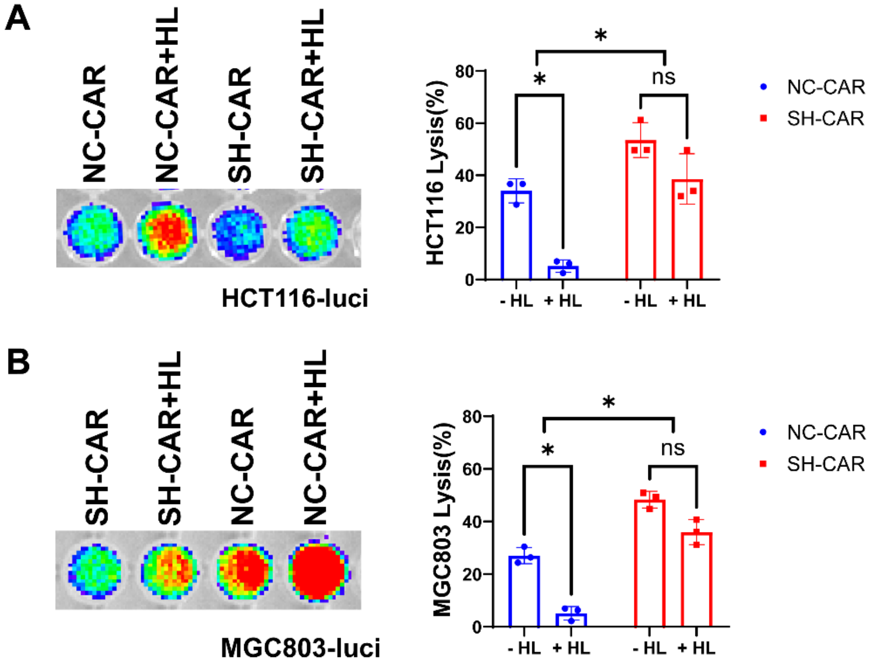

Fig. S19. Downregulation of GPR132 attenuates the inhibition of CAR-NK cell function by lactate. Bioluminescence imaging was employed to assess the HCT116-luciferase (**A**) and MGC803-luciferase (**B**) cells after 4 hours co-cultured with NC-CAR-NK92 or SH-CAR-NK92 cells at 5: 1 effector-to-target (E: T) ratios. NC-CAR-NK92 and SH-CAR-NK92 cells were treated with lactate for 12 hours or not. The fluorescence image is shown on the left, and the quantified killing rate is shown on the right. The experiment was performed three times. Data are shown as mean ± SD and were analyzed by two-way ANOVA. (*$P < 0.05$, ns, not significant)

Fig. S20.

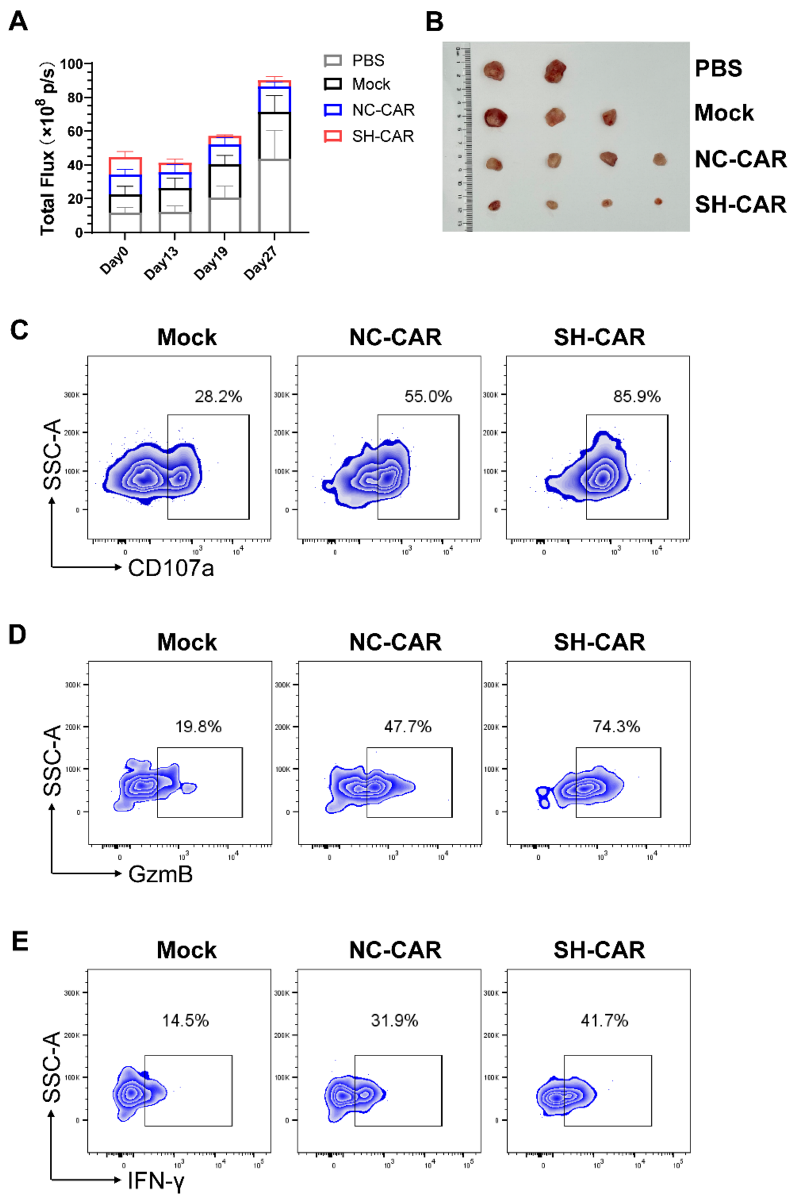

Fig. S20. Downregulation of GPR132 enhances the ability of CAR-NK92 cells against colorectal cancer in vivo. (A) The bar columns show the total bioluminescence flux (photons per second) of the tumor on days 0, 13, 19, and 27 (n=4). **(B)** Tumors were retrieved from the mice on day 35 and subjected to digital imaging (n=4). **(C-E)** The representative FACS profile displays CD107a, GzmB, and IFN-γ expression in $CD45^{+}CD56^{+}$ cells from tumors (n=3), which corresponds to the column chart of **Figures 8G, H,** and **I**, respectively.

Fig. S21.

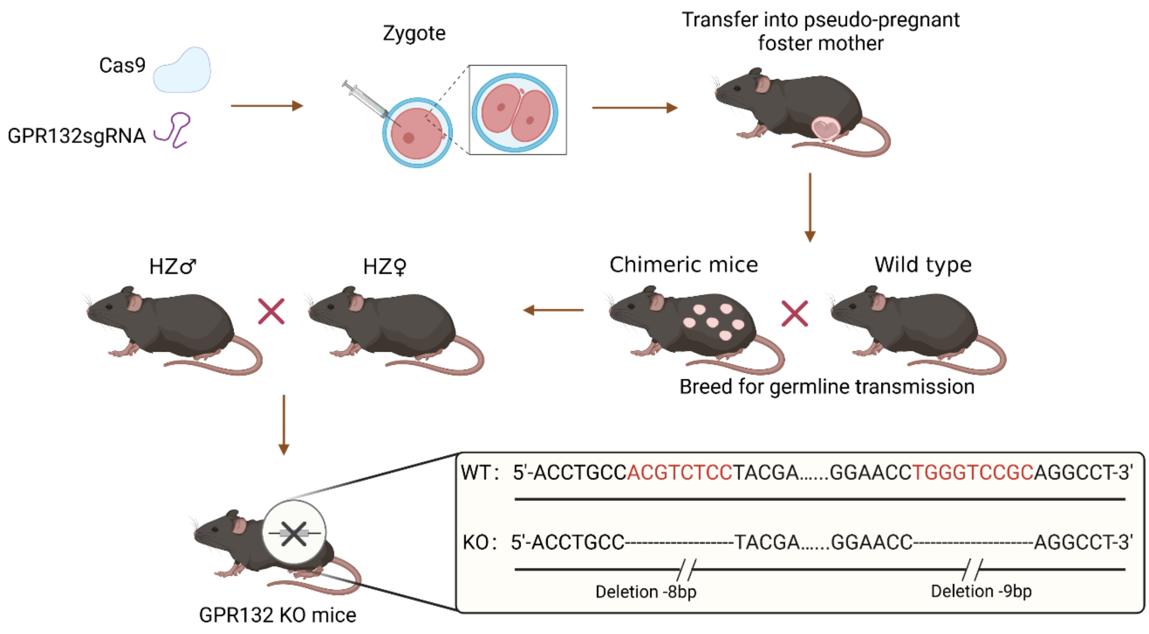

Fig. S21. Graphical abstract shows the development of GPR132 Global KO mice.

Table S1.

Table S1: List of shRNA sequences of GPR132

Name	Sequences	GC (%)
shRNA1	5'-GGGTCATCTATATCCGCAACC-3'	52%
shRNA2	5'-GGTACTACTACGCCAGGTTCA-3'	52%
shRNA3	5'-GGTGGAAAGAGTGGTCCATGA-3'	52%
shRNANC	5'-GGATCCATAGTCGTGGTAATC-3'	48%

Table S2.

Table S2: List of primers for RT-PCR

Gene Name	Sequences
GPR132	Forward 5'-ATCCTCTTCCTGTGCTGCAT-3'
	Reverse 5'-GCTTTGACGAGGAGAACCAG-3'
Gαs	Forward 5'-GACTTTGACTTCCCTCCCGA-3'
	Reverse 5'-CAAGGACTTTCTCAGCGAGC-3'
Gαi1	Forward 5'-GATGATGCACGCCAACTCTT-3'
	Reverse 5'-TTCAGCTAGAACCAGGTCGT-3'
Gαi2	Forward 5'-ACGACTCAGCTGCCTACTAC-3'
	Reverse 5'-ACTCAGGGAAGCAGATGGTC-3'
Gαi3	Forward 5'-TGGGACGGCTAAAGATTGACT-3'
	Reverse 5'-GCATTCGGTTCATCTCCTCG-3'
β-actin	Forward 5'- GTACGCCAACACAGTGCTG-3'
	Reverse 5'-CGTCATACTCCTGCTTGCTG-3'

Table S3.

Table S3: List of antibodies used to perform Flow cytometry

**Antibody Name**	**Company**	**Clone**	**Catalog number**
PE Anti-mouse CD3	BD Biosciences	17A2	555275
APC Anti-mouse CD4	Biolegend	GK1.5	100412
BV421 Anti-mouse CD8a	Biolegend	53-6.7	100753
PE Anti-mouse NK1.1	Biolegend	PK136	108707
APC Anti-mouse NK1.1	Biolegend	PK136	108709
APC Anti-mouse B220	Biolegend	RA3-6B2	103211
APC Anti-mouse CD11c	Biolegend	N418	117309
FITC Anti-mouse CD11b	BD Pharmingen	M1/70	553310
FITC Anti-mouse CD86	Biolegend	RL388	128207
APC Anti-mouse F4/80	Biolegend	BM8	123115
PE Anti-mouse CD107a	Biolegend	1D4B	121611
PE/Cy7 Anti-mouse GzmB	eBioscience	NGZB	4310223
APC Anti-mouse IFN-γ	Invitrogen	XMG1.2	2410273
APC Anti-human CD69	Biolegend	FN50	310910
PE/Cy7 Anti-human CD107a	Biolegend	H4A3	328618
PE/Cy7 Anti-human GzmB	Biolegend	QA16A02	372214
APC Anti-human IFN-γ	Biolegend	4S. B3	502512
APC Anti-human Ki67	Biolegend	Ki-67	350513
Alexa Fluor 647 Anti-human Bcl-2	Biolegend	100	658708
Rabbit monoclonal Anti CSK	Abcam	EPR24673-97	ab300132
Alexa Fluor 647Anti-rabbit IgG	CST	/	4414S
PE Anti-human ZAP70	Biolegend	A16043E	396003
PE Anti-human NF-κB	BD Biosciences	K10-895.12.30	558423
APC Anti-human NKG2D	BD Biosciences	1D11	558071
PE Anti-human MICA/B	Biolegend	6D4	320906
APC Annexin V	Biolegend	/	640920

Original FACS data

- **Figure 2G**

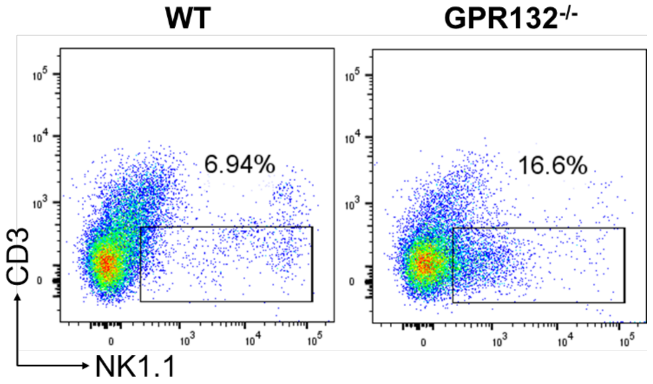

- **Figure 2H**

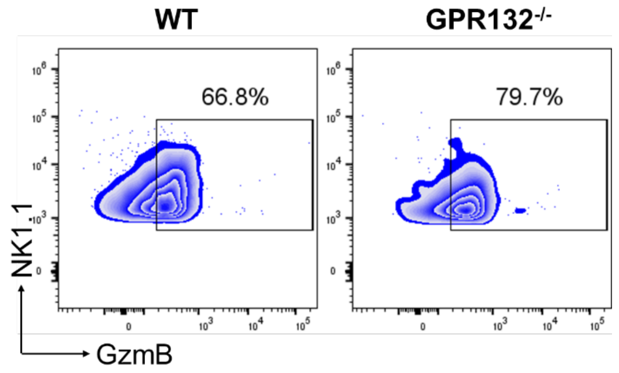

- **Figure 2I**

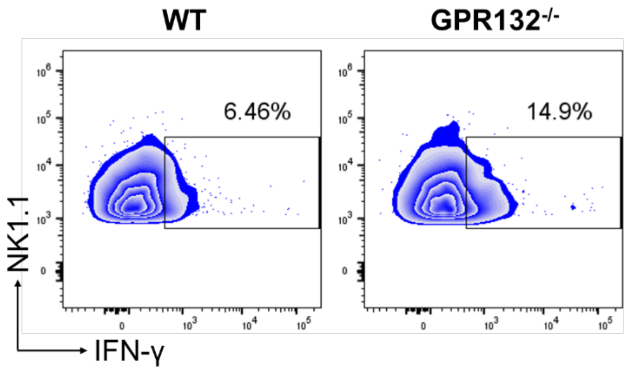

- **Figure 3A**

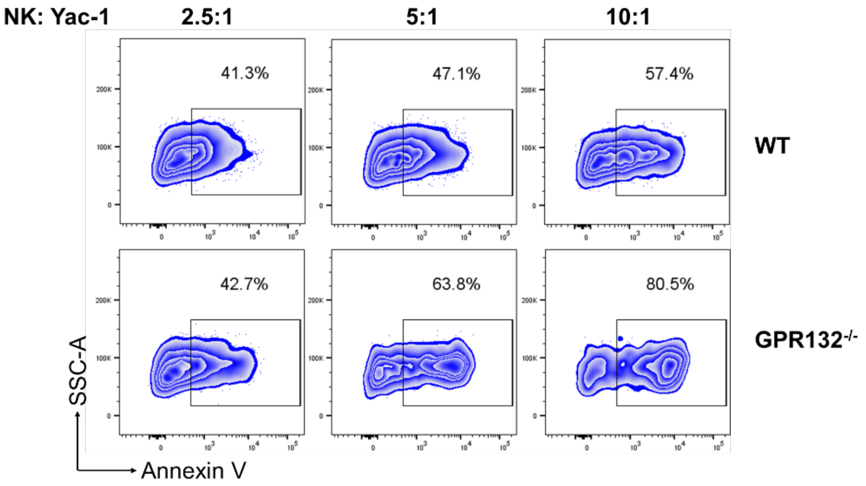

- **Figure 3B**

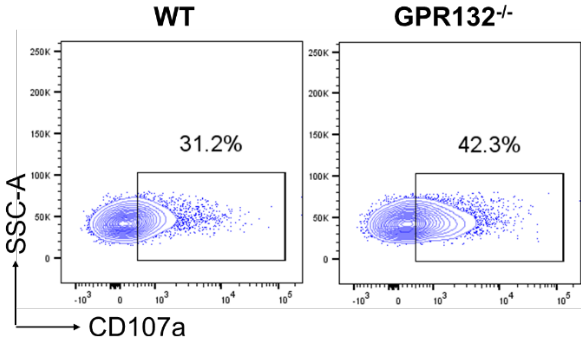

- **Figure 3C**

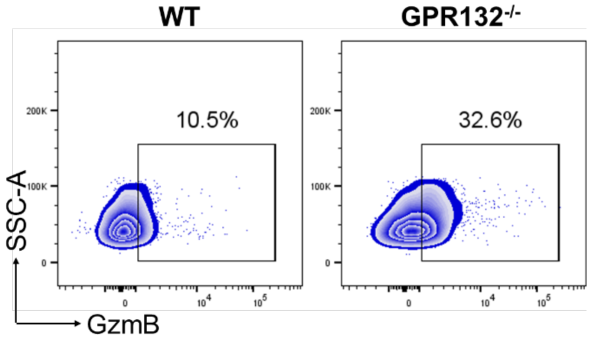

- **Figure 3D**

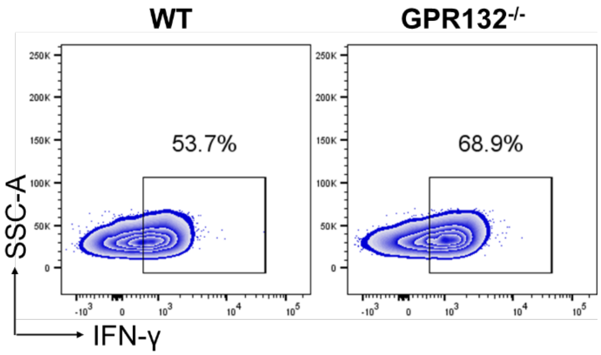

- **Figure 3G**

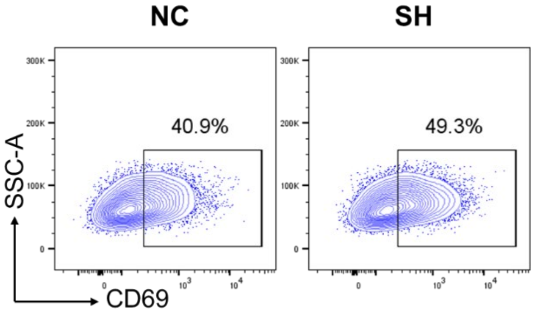

- **Figure 3I**

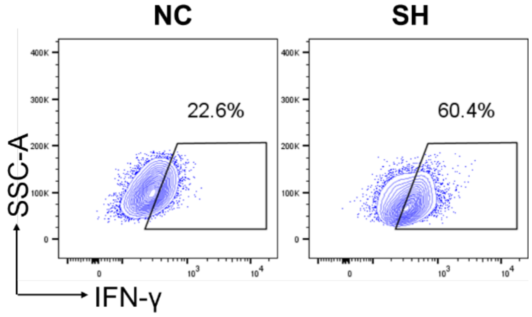

Original gel image

1. Original nucleic acid electropherogram

- **Figure 1D**

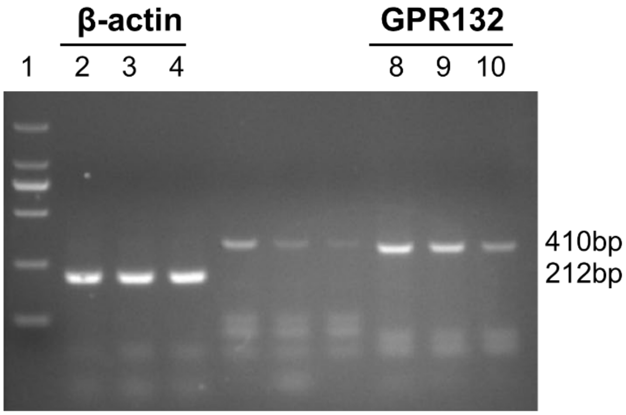

1: DL2000 Marker;
2, 8: 0h;
3, 9: 6h;
4, 10: 12h.

- **Figure S6C**

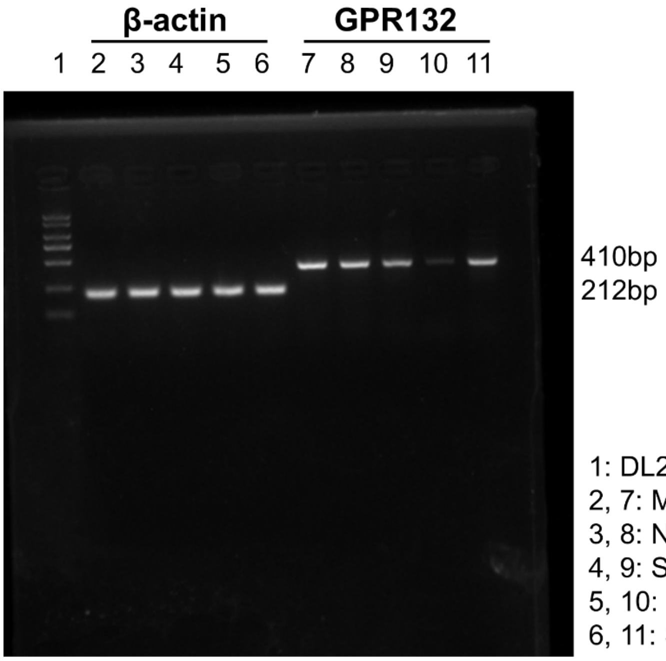

1: DL2000 plus Marker;
2, 7: Mock-NK92;
3, 8: NC-NK92;
4, 9: SH1-NK92;
5, 10: SH2-NK92;
6, 11: SH3-NK92.

- **Figure S16A**

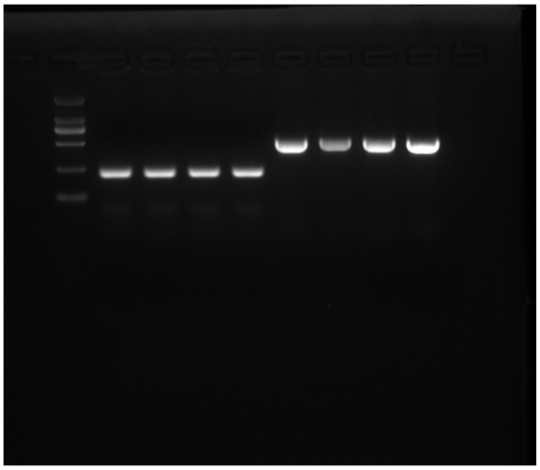

1: DL2000 Marker;
2, 6: NC-NK92;
3, 7: SH-NK92;
4, 8: Control-NK92;
5, 9: ONC212-NK92

- **Figure S16B**

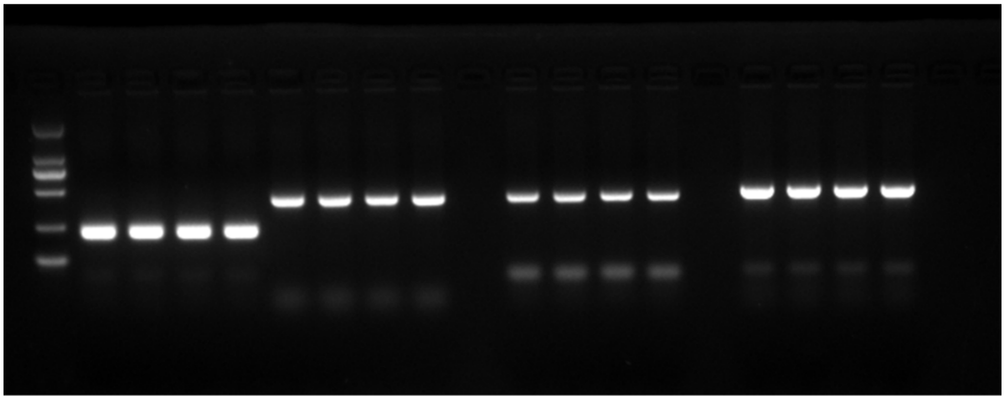

β-actin: 212bp Gαi1: 405bp Gαi2: 423bp Gαi3: 468bp

1: DL2000 Marker;
2, 6,10,14: NC-NK92;
3, 7,11,15: SH-NK92;
4, 8,12,16: Control-NK92;
5, 9,13,17: ONC212-NK92

- **Figure S17C**

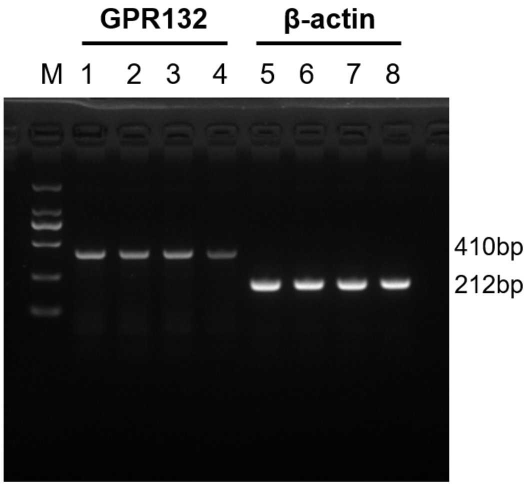

M: DL2000 Marker;
1, 5: Mock-NK92;
2, 6: CAR-NK92;
3, 7: NC-CAR-NK92;
4, 8: SH-CAR-NK92.

2. Original Western Blot

- **Figure 1E**

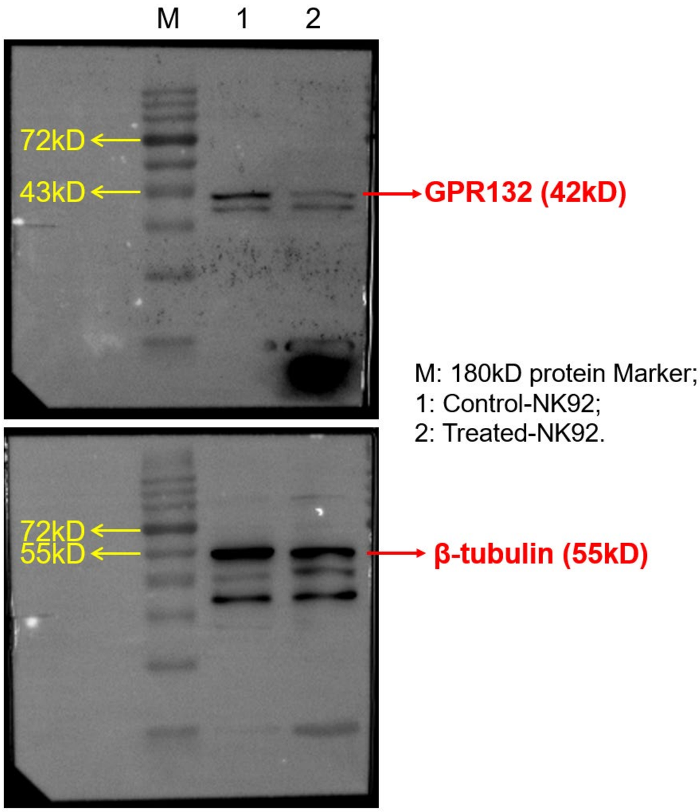

M: 180kD protein Marker;
1: Control-NK92;
2: Treated-NK92.

- **Figure S6D**

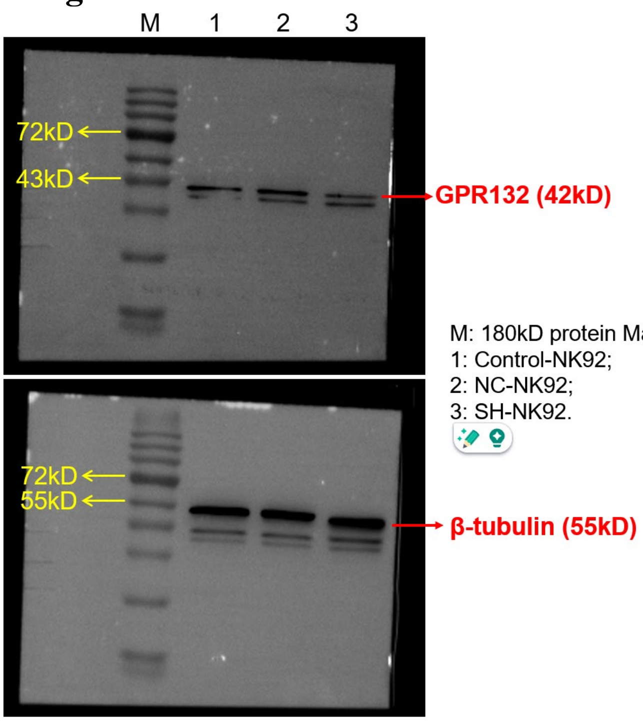

M: 180kD protein Marker;
1: Control-NK92;
2: NC-NK92;
3: SH-NK92.

- **Figure S17D**

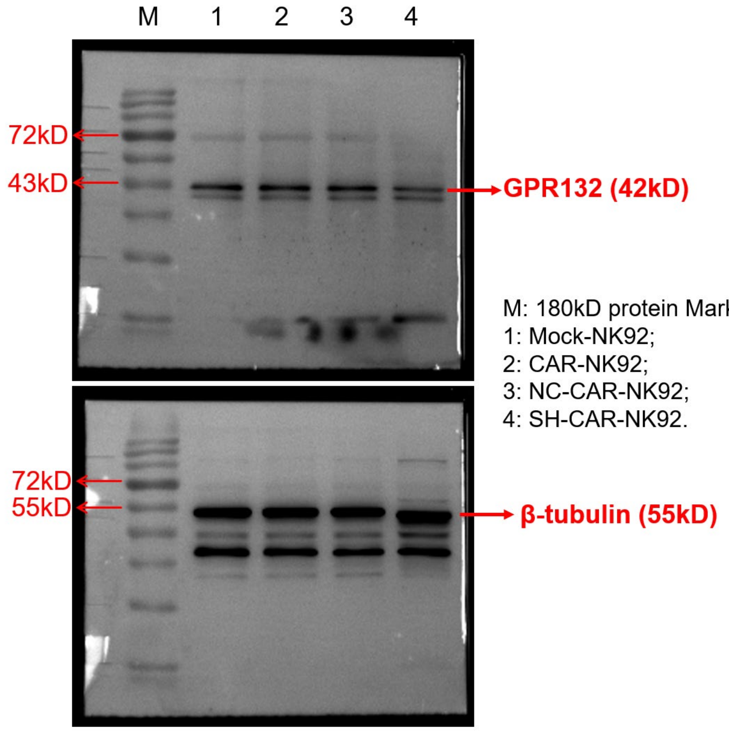

M: 180kD protein Marker;
1: Mock-NK92;
2: CAR-NK92;
3: NC-CAR-NK92;
4: SH-CAR-NK92.
